# Helping authors produce FAIR taxonomic data: evaluation of an author-driven phenotype data production prototype

**DOI:** 10.1093/database/baae097

**Published:** 2025-01-29

**Authors:** Limin Zhang, Julian Starr, Bruce Ford, Anton Reznicek, Yuxuan Zhou, Étienne Léveillé-Bourret, Étienne Lacroix-Carignan, Jacques Cayouette, Tyler W Smith, Donald Sutherland, Paul Catling, Jeffery M Saarela, Hong Cui, James Macklin

**Affiliations:** School of Information, University of Arizona, 1103 E. 2nd Street, Tucson, AZ 85719, USA; School of Fine Arts, Huaiyin Normal University, 71 Jiaotong Road, Huaian, Jiangsu 223001, China; Department of Biology, University of Ottawa, 30 Marie Curie, Ottawa, ON K1N 6N5, Canada; Department of Biological Sciences, University of Manitoba, 50 Sifton Road, Winnipeg, MB R3T 2N2, Canada; University Herbarium, University of Michigan, 3600 Varsity Drive, Ann Arbor, MI 48108, US; School of Information, University of Arizona, 1103 E. 2nd Street, Tucson, AZ 85719, USA; Department of Biological Sciences, Université de Montréal, 1375 Avenue Thérèse-Lavoie-Roux, Montréal, QC H3A 2B3, Canada; Department of Biological Sciences, Université de Montréal, 1375 Avenue Thérèse-Lavoie-Roux, Montréal, QC H3A 2B3, Canada; Research and Development Centre, Agriculture and Agri-Food Canada, 960 Carling Avenue, Ottawa, ON CA K1A 0C6, Canada; Research and Development Centre, Agriculture and Agri-Food Canada, 960 Carling Avenue, Ottawa, ON CA K1A 0C6, Canada; Natural Heritage Information Centre, Ontario Ministry of Natural Resources, P.O. Box 7000, Peterborough, Ontario K9J 8M5, Canada; Research and Development Centre, Agriculture and Agri-Food Canada, 960 Carling Avenue, Ottawa, ON CA K1A 0C6, Canada; Research and Collections, Canadian Museum of Nature, 240 McLeod St, Ottawa, ON K1P 6P4, Canada; School of Information, University of Arizona, 1103 E. 2nd Street, Tucson, AZ 85719, USA; Research and Development Centre, Agriculture and Agri-Food Canada, 960 Carling Avenue, Ottawa, ON CA K1A 0C6, Canada

## Abstract

It is well-known that the use of vocabulary in phenotype treatments is often inconsistent. An earlier survey of biologists who create or use phenotypic characters revealed that this lack of standardization leads to ambiguities, frustrating both the consumers and producers of phenotypic data. Such ambiguities are challenging for biologists, and more so for Artificial Intelligence, to resolve. That survey also indicated a strong interest in a new authoring workflow supported by ontologies to ensure published phenotype data are FAIR (Findable, Accessible, Interoperable, and Reusable) and suitable for large-scale computational analyses.

In this article, we introduce a prototype software system designed for authors to produce computational phenotype data. This platform includes a web-based, ontology-enhanced editor for taxonomic characters (Character Recorder), an Ontology Backend holding standardized vocabulary (the Cared Ontology), and a mobile application for resolving ontological conflicts (Conflict Resolver). We present two formal user evaluations of Character Recorder, the main interface authors would interact with to produce FAIR data. The evaluations were conducted with undergraduate biology students and *Carex* experts. We evaluated Character Recorder against Microsoft Excel on their effectiveness, efficiency, and the cognitive demands of the users in producing computable taxon-by-character matrices.

The evaluations showed that Character Recorder is quickly learnable for both student and professional participants, with its cognitive demand comparable to Excel’s. Participants agreed that the quality of the data Character Recorder yielded was superior. Students praised Character Recorder’s educational value, while *Carex* experts were keen to recommend it and help evolve it from a prototype into a comprehensive tool. Feature improvements recommended by expert participants have been implemented after the evaluation.

## Introduction

In published literature, phenotypic data often take two main forms, one is taxon-by-character matrices, and the other is morphological/phenotypic descriptions. It is well-known that the use of vocabularies in these publications is often inconsistent. While natural language processing and machine learning techniques are capable of extracting phenotypic information from the literature [e.g. 1, 2], due to the significant variability in vocabulary usages [e.g. 3, 4] and their lack of clear definitions, the extracted characters and character states do not have the interoperability and reusability for broader biological research [5, 6].

Further, translating extracted raw character data into ontological expressions is also extremely challenging. Not only computerized methods underperformed human data curators in conversion accuracy, the variation among human curators themselves was also remarkable, sometimes exceeding 40% [5]. Inter-curator variation is widely reported by other projects in biology and other domains [4, 7–11]. This variability is influenced by factors such as the curators’ academic background, the original descriptions’ clarity, issues with ontology usability, and the inherent creativity of human intelligence. Additionally, the studies done by the Phenoscape project revealed that even the consensus reached by multiple curators does not always align with the original authors’ intentions, highlighting the complexities in post-publication phenotypic data curation [3].

These findings suggest that neither machine learning, including large language models used by Generative AI, nor professional curation [12] stands as sustainable solutions for FAIR phenotype data (Findable, Accessible, Interoperable, and Reusable) production. Interoperability and reusability in FAIR depend on well-defined data that can be computationally and reliably compared across projects/domains. However, current publications often include unstandardized descriptors—”maroon” versus “red brown,” “lanceolate” versus “linear,” and differing measurements such as “3mm” to “5mm,” which cannot be reliably compared without knowing the landmarks used for the measurements. While these may be clear to an individual researcher or within a project, once published, the connections to these local definitions are often lost. Such ambiguity in published phenotypic characters has frustrated data producers and data consumers alike, as revealed by a recent survey [13]. Among the respondents, 73% were frustrated with the ambiguity, and 85% were willing to adopt a new authoring workflow to produce FAIR phenotypic data for publication.

Authors hold sole authority over the data they produce [14]. In the “Authors in the Driver’s Seat” project, we developed a prototype software system to explore the integration of ontologies into the taxonomic data authoring workflow. This proof-of-concept system allows for characters and character states to be defined using ontologies, enabling the harvesting of the taxon-by-character matrix as both knowledge graphs (e.g. RDF, Resource Description Framework) for computer agents and text narratives for human readers.

**Figure 1.**
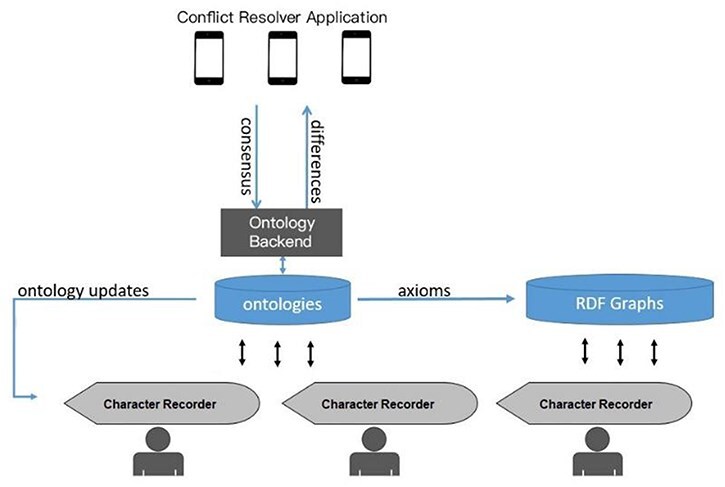
System diagram for the author-driven computable phenotypic data and ontology development platform.

The prototype system consists of three main components, Character Recorder, Ontology Backend, and Conflict Resolver (Fig. 1). The web-based Character Recorder provides an interface for creating character matrices with ontology terms and communicates with the Ontology Backend. The Ontology Backend (https://github.com/biosemantics/charaparser-web) is a customized Java Webservice employing Java Owl API. It loads Carex Ontology, and therefore holds all characters, character states, and the necessary concepts for their definitions and for Character Recorder semantic functionality. The Ontology Backend receives new terms from Character Recorder users and monitors ontologies for potential issues, which are then forwarded to the mobile app, Conflict Resolver. This app allows domain experts to address and resolve ontological issues, and these resolutions are fed back to the Ontology Backend. Users of the Character Recorder are notified of ontology updates via its matrix and ontology updates interfaces. This workflow enables users to: (i) immediately add and utilize new terms when needed, (ii) remain informed about updates to their primary domain ontologies, and (iii) contribute to the ontologies’ development to ensure they align with their cognitive framework. As a case study, we used the plant genus *Carex* and created the Carex Ontology with relevant semantic constructs needed to support Character Recorder. While the Ontology Backend can load any OWL ontologies, the Carex Ontology provides the specific annotations and relations needed to support the semantic features of the Character Recorder.

In this article, we describe the construction of Carex Ontology and report two usability studies of Character Recorder, which is the primary interface authors would interact with. These studies of Character Recorder involved undergraduate biology students and professional taxonomists. We examined the effectiveness and efficiency of its features and the cognitive effort required of the users, and compared them with Microsoft Excel, which remains the most popular tool for editing taxon-by-character matrices. We hope our design of the Ontology and findings on the usefulness of the software features will provide useful insights for the interested communities to further develop Character Recorder and other ontology-enhanced data production tools.

## Materials and Methods

### Materials: The Carex Ontology

The Carex Ontology is the primary ontology utilized in the prototype, alongside the Modifier Ontology [15]. The creation of the Carex Ontology involved the following steps:

1. Candidate phenotype terms were extracted from the *Carex* treatments in the Flora of North American V.23 (Cyperaceae) using the Explorer of Taxon Concepts (ETC) Text Capture tool [2]. ETC Ontology Builder was then used to produce a raw ontology based on the extracted characters and character states. Access to ETC tools is available at http://etc.sbs.arizona.edu/etcsite/.
2. Terms irrelevant to *Carex* phenotypes were manually removed from the raw ontology by an undergraduate research assistant supervised by a *Carex* taxonomist. The assistant also adjusted subclass relations among the terms.
3. The raw ontology was then imported to WebProtege (https://webprotege.stanford.edu/). At this time, the two top-level main classes were “anatomical entity,” the superclass for all anatomical structures, and “quality,” the superclass for all character and character state terms.
4. In WebProtege, the raw ontology was further refined by adding appropriate annotation properties and object properties:
  - We synonymized various expressions of the same concepts using obo:exact_synonyms Annotations such as obo:hasBroadSynonym, and carex:has_not_recommended_synonym were used to associate a term with other related words. For instance, carex:acute, denoting a shape, has a not recommended synonym of “acutish,” indicating the latter is imprecise and discouraged for use. Similarly, carex:bright, describing color brightness, has a broad synonym “light,” signifying carex:bright corresponds to only one meaning of “light.” Terms frequently used in the literature but considered suboptimal by Carex experts were marked as obsoleted. The carex:term_replaced_by annotations guide users to preferred terms For example, carex:awl_shaped has been deprecated in favor of carex:subulate.
  - We added annotation properties, such as oai: elucidation, which link to graphic illustrations of specific characters or character states. We also introduced object properties, including carex:measured_from, carex:measured_to, carex: measured_include, carex: measured_exclude, and carex:measured_at, to detail the reference points used in numerical measurements of characters. For the measurement units and scopes, we added a unit class and a scope class. Furthermore, the carex:in_collection property was incorporated to link characters to their respective collections, such as the carex: carex_standard_character_set.
  - We incorporated a set of core characters, identified by *Carex* experts on the project, into the Ontology. These are essential for any description of a *Carex* taxon and recognized as the “Recommended Set of Characters” in Character Recorder and the carex: carex_standard_character_set in the Carex Ontology.
  - We introduced a carex: to review class within the Ontology to accommodate new term suggestions from Character Recorder’s end-users. Upon expert review via the Conflict Resolver, these user-contributed terms are either integrated into the Ontology under the appropriate category (using rdfs:subclassOf) or deprecated in favor of a replacement term. Notifications about these updates and other ontological changes are available on the Character Recorder’s Ontology Updates page. Moreover, in matrices, data cells containing deprecated terms are marked with small wrench icons to signal that users need to review and possibly accept the suggested replacement terms Importantly, to maintain the Ontology’s and the matrices’ stability, the IDs of terms are kept persistent regardless of changes.
  - Lastly, for terms with equivalent concepts in other ontologies (such as the Plant Ontology and the Relation Ontology), we added cross-references to enhance interoperability.

**Figure 2.**
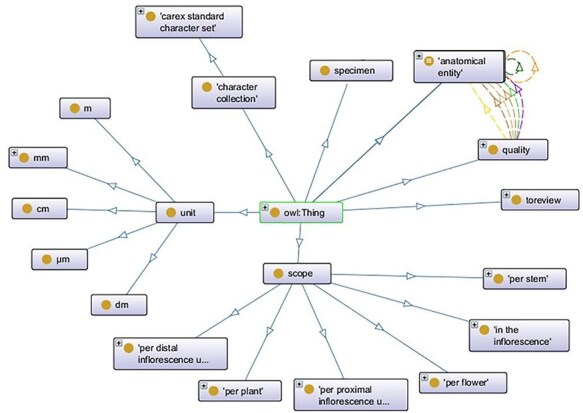
Top-level classes in the Carex Ontology.

The Carex Ontology used in the experiments contained 1880 classes, 44 object properties, 34 annotation properties, and a variety of axioms (2902 class axioms, 74 object property axioms, and 6768 annotation axioms). Top-level classes are shown in Fig. 2. Among the top-level classes, obo:anatomical_entity and carex: quality encompasses the majority of terms within the ontology, where quality terms are linked to entity terms through properties, such as carex:measured_from, carex:measured_to, carex: includes, carex: excludes, and carex:measured_at. Additionally, associations between entity terms are facilitated by the carex:maybe_part_of and carex:join_with properties.

### Materials: Character Recorder

The design of Character Recorder was primarily based on domain scientists’ input and benefited from a few earlier studies: (i) The Create Character function in Character Recorder was evolved from the Measurement Recorder, which enables users to define new numerical characters for the Ontology for subsequent use in matrices [16]. (ii) The user interface of Character Recorder for adding terms to the Ontology was developed based on findings from Reference [17]. (iii) Moreover, applying data mining methods on 2283 colors extracted from high-quality *Carex* specimen images, color palettes for brown, yellow–brown, yellow–green, green, and red were created and employed in Character Recorder [18]. These palettes allow users to record colors directly, rather than selecting or creating color names to reduce the subjectivity associated with color naming.

**Figure 3.**
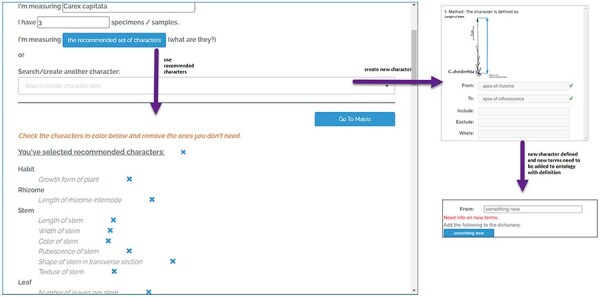
The character setup page of Character Recorder, accessible after logging in.

**Figure 4.**
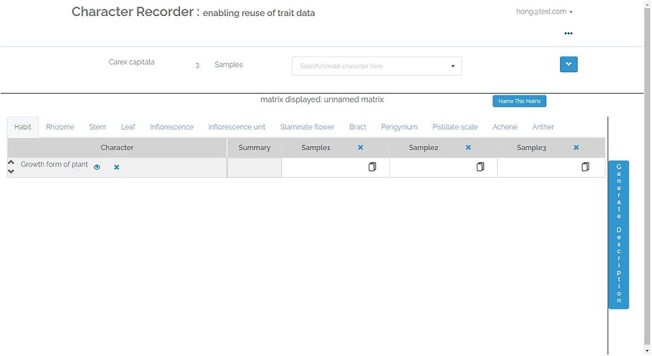
The matrix page of Character Recorder.

The Character Recorder prototype integrated many features to guide users to select and use appropriate terms from the Carex Ontology in their character matrices. Figures 3–7 explain a few key features of the Character Recorder version used in the studies reported here. Improvements to these features have been implemented based on the findings from the studies and are described later.

As shown in Figure 3, users can employ the “Recommended Set of Characters” or create new characters for a matrix. The “Recommended Set of Characters” constitutes a minimal set of core characters from the Carex Ontology. Each Carex treatment should include this set to ensure parallelism in descriptions. A prominent button prompts users to utilize the recommended set, but they also have the option to add specialized characters. This can be done by searching for existing characters known to the platform or by creating new ones. When adding a new numerical measurement character, users must define the reference points for the measurements. This requires selecting existing terms from the Ontology or contributing new terms Reference points that are recognized by the Ontology are marked with green checkmarks, indicating approval.

Figure 4, shows the matrix page. The matrix, containing user-selected characters, appears empty initially. The characters related to various organs are arranged across different tabs. The “Generate Description” button can be clicked to generate a textual description of the taxon based on the matrix’s data. Following this, an “Export” button becomes accessible, enabling the user to export the matrix as a CSV file, the description as a DOC file, and RDF triples as a TRIG file. Refer to Figure 5 for an overview of the features that support the formulation of character.

As Figure 5 shows, the input template assists the user in formulating a character state, allowing for free-text pre- and post-constraints and the incorporation of certainty and degree modifiers from the Modifier Ontology. Top part of Figure 5 shows that when the user clicks in an entry box associated with a character (e.g. growth form), the corresponding branch of the ontology opens for the user to select a term. The image icon or color icon next to a term can be evoked to open illustrations associated with the term or relevant color palettes. The user can also enter a value directly (“cespitose” in this case). The platform searches in the Carex Ontology for matches and permits the addition of new terms to the Ontology. In this example, with the user’s approval, “cespitose” is replaced with its preferred term “caespitose.” Any new term contributed by users is immediately incorporated into the Carex Ontology and submitted to the Conflict Resolver for review. Character states previously recorded by other users for the same taxon and character are available, enabling reuse by the current user. The lower two screenshots of Figure 5 show that when an illustration (or a color palette) for a character state is available in the ontology, the user can click on the corresponding icon to invoke the illustration or color palette. When the user selects a color in a palette, the system record the sRGB values and the color label (eg., ‘light-yellow brown’) for the color.

**Figure 5.**
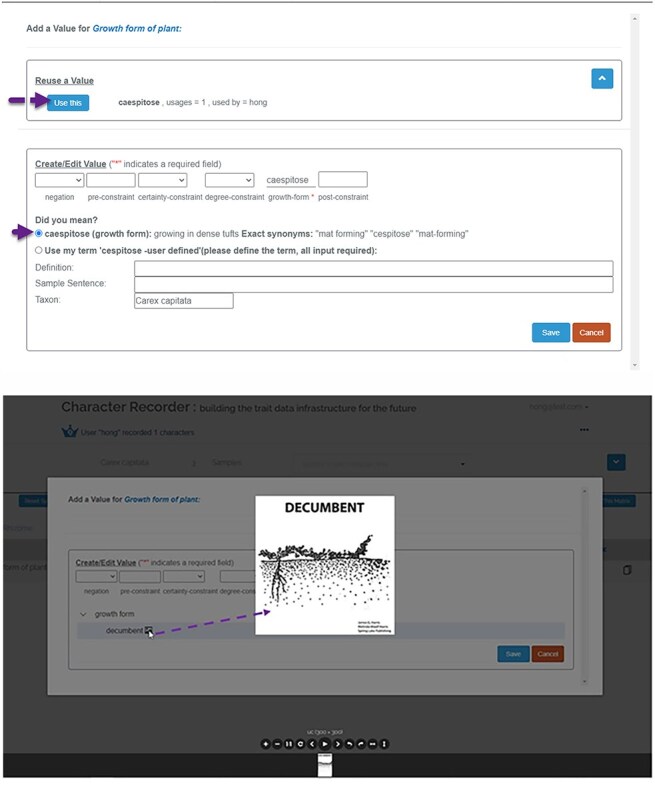


**Figure 5.**
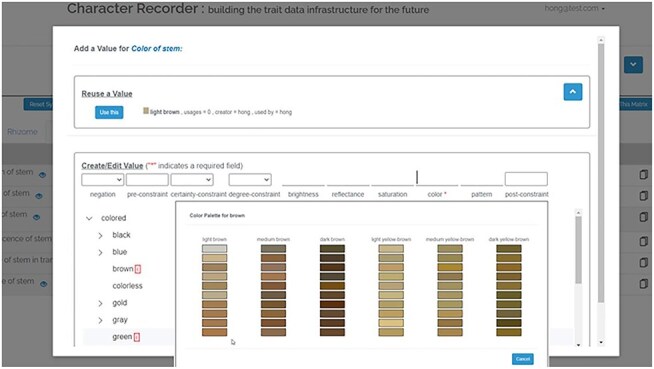
The Character State Window, accessible when the user clicks on any data cell of a categorical character.

**Figure 6.**
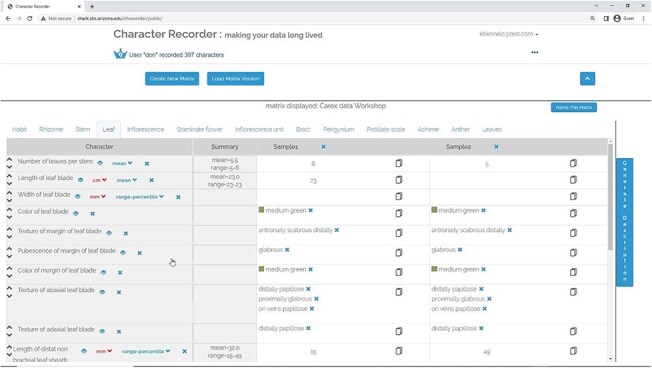
The Matrix Page. A portion of the matrix related to leaves is filled out.

**Figure 7.**
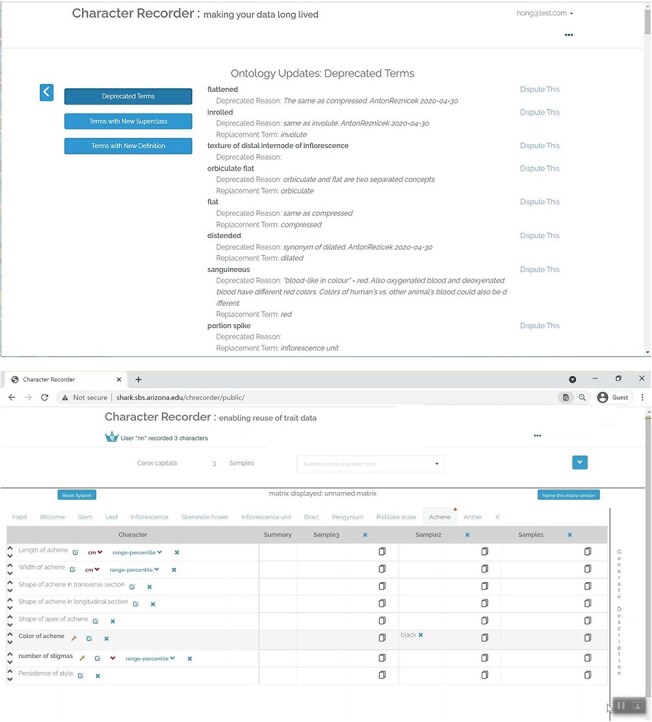
Ontology updates in Character Recorder.

The top screenshot in Figure 7 shows Character Recorder’s Ontology Updates Page, which outlines three types of updates, with the depicted section focusing on the list of deprecated terms. Users are provided with an option to contest a term’s deprecation. The lower screenshot in Figure 7 shows that, when terms used in existing matrices become deprecated, users are notified during matrix review through an orange dot appearing next to the name of the tab containing deprecated terms. In the tab,the deprecated characters or character states are marked with an orange wrench icon. By clicking on this icon, users can choose to either accept the recommended replacement term or challenge the deprecation.

### Methods: usability studies of Character Recorder

Two usability experiments were conducted on the Character Recorder, each with distinct emphases but sharing some commonalities. The first study referred to as the Student Experiment, engaged undergraduate biology students to evaluate the overall usability of the features. The second study, known as the Expert Experiment, involved professionally trained botanists specializing in *Carex* taxonomy and/or the identification of *Carex* specimens. The focus of the Expert Experiment was on the effectiveness of the workflow design, ontology integration, character illustrations, and color palettes, as well as the dynamics of character reuse among experts. Given that MS Excel is the most widely used tool for editing character matrices, both studies compared Character Recorder with Excel in terms of effectiveness, efficiency (including cognitive demand), and user satisfaction in recording FAIR data. Both studies received Institutional Review Boards’ (IRB) approvals from relevant authorities (University of Arizona protocol # 2105767649; University of Manitoba protocol #: HE2021-0066; and University of Ottawa protocol# H-07-21-7202).

#### Student experiment

A total of 16 undergraduate students from the Biology Departments of the University of Manitoba (13) and the University of Ottawa (3) participated in the online usability study of Character Recorder from November 2021 to January 2022. Prior to the study, all students had completed a module on *Carex* morphology in their Plant Biology courses.

Participants were assigned unique IDs and followed a standardized procedure that began with a demographic questionnaire (Supplementary Appendix A). This was followed by a self-paced training session on Character Recorder (Supplementary Appendix B) and a hands-on task session where participants recorded six characters provided to them for each of the two samples of a *Carex* taxon, using Character Recorder and Excel in different orders (Supplementary Appendix C). A session concluded with a user experience questionnaire (Supplementary Appendix D). There was no interaction among participants throughout the session. Each participant received a $20 Amazon gift card upon completion of a session that lasted 60 min or less.

To control for learning effects, half of the participants were randomly assigned to start with Excel and then use Character Recorder, while the other half followed the reverse order. The study was conducted over Zoom, with screen activities recorded for analysis, alongside the data recorded in the Character Recorder and participants’ questionnaire responses.

#### Expert experiment

We sent invitations to 15 *Carex* systematists residing in the United States or Canada and a total of 9 *Carex* experts, each with at least one taxonomic publication on *Carex*, agreed to take part in the experiment. This 3-day experiment took place in person at a fully equipped biology lab at the University of Ottawa in April 2022.

Experts were assigned unique participant IDs and followed an overall procedure similar to that of the student participants but less structured: Firstly, the self-guided training session in Student Experiment was replaced by a 10-min live demonstration of Character Recorder, followed by a 30-min Q&A session. Secondly, the hands-on task session for experts was designed to be life-like and unstructured, extending over 2 days, as opposed to the students’ task of recording a few given characters in less than 1 h. Experts used desktop computers running Character Recorder to record relevant characters from mounted specimen sheets of *Carex canescens* L. and *Carex rostrata* Stokes. These species were chosen because of their contrasting phenotypes (e.g. unisexual versus bisexual inflorescence units, differences in perigynium and achene morphology, etc.) thus accentuating the range of variation found within the genus. Discussions among experts and with the experimenter in the hands-on session were allowed and encouraged. Although the experts’ activities were not video recorded, the character matrices they generated and their verbal feedback on the software were meticulously documented. Thirdly, the experts’ hands-on session was followed by a 1-day focused discussion session, addressing issues identified during the first 2 days and exploring potential solutions. Lastly, the user experience questionnaire experts independently and anonymously filled out contained a few additional questions on their overall assessment of the Character Recorder. For their active participation, each expert received a $50 Amazon gift card for every half-day of the experiment.

Note, unlike the Student Experiment, the experts exclusively used Character Recorder, foregoing Excel. This decision was based on the expert’s familiarity with Excel, which allowed them to compare the two without necessitating a direct engagement with Excel.

#### Scoring of participant responses to user experience questionnaire

All questions in the user experience questionnaire were constructed using Likert scales ranging from 3 to 9 points. Participant responses were scored according to the respective scale, and the final score for each question is the average over all responses to the question. For instance, for a question employing a 3-point Likert scale, such as “[the component] did what I wanted it to do,” a response of “rarely” was scored as 1, “sometimes” as 2, and “always” as 3. The final score for the question was calculated as the sum of all scores divided by the total number of responses. This average score is interpretable on the original scale, for example, a mean of 2.8, which lies between “sometimes” and “always” but leans closer to “always,” can be interpreted as “almost always.”

## Results and analyses

All 16 student participants and 8 out of 9 expert participants completed the entire experiment sessions including both questionnaires. One expert participant departed early and did not answer the user experience questionnaire.

### Demographic results

Fourteen undergraduate and two master’s students participated in the Student Experiment. The group consisted of 13 females, 2 males, and 1 non-binary individual. All students had limited knowledge about *Carex*, having been introduced to *Carex* morphology only in an undergraduate course. The nine expert participants were all male and possessed substantial knowledge of *Carex*. They were at different stages of their careers and ranged in age from 20 to over 69 years. Among them, 3 participants were aged between 20–49 years, 2 were between 50 and 59 years, and 4 were older than 60. The age group boundaries were created to correspond to career stages, establishing scientists, established scientists, and retiring/retired but still active scientists.

### Effectiveness of Character Recorder: software features and student data quality

To assess the extent to which participants could effectively use Character Recorder to record FAIR characters, we analyzed their responses to the questions relevant to software effectiveness in the user experience questionnaire. We also reviewed the quality of the character data students recorded.

**Figure 8.**
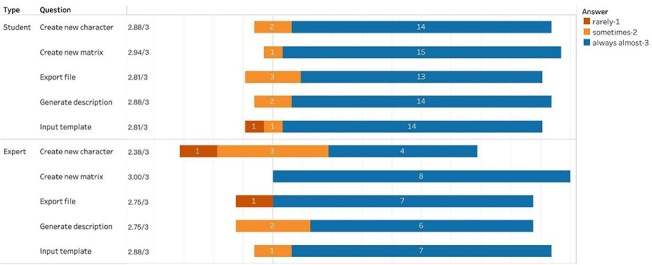
Summary of the responses to “Your experience with the main components of Character Recorder: - Did what I wanted it to do.”

Figure 8 summarizes participants’ scores on the effectiveness of all five functional components of the Character Recorder: *Input Template, Create New Character, Create New Matrix, Export File*, and *Generate Description*. Numbers in the bars are response counts, while the colors denote participant answers. The average score is noted to the left of each bar. The vertical line going through all bars indicates the middle (neutral) position, or “sometimes” in this case. The fact that all bars extend far to the right of the neutral line indicates a strong agreement towards “almost always.” The students’ average scores on these components were 2.8 or above, while the experts’ average scores for all but *Create New Character* were 2.75 or above. These results indicate that the Character Recorder is effective in supporting participants’ character recording tasks. The need for improvement was identified in creating novel characters for phylogenetic research, and expert-suggested improvements were implemented after the experiment.

**Figure 9.**
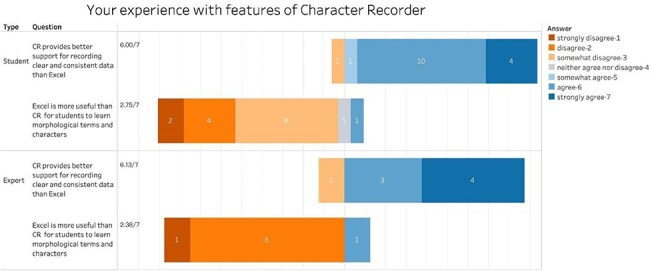
Summary of the responses to “Your experience with features of Character Recorder” in recording clear characters compared to Excel.

Participants were also asked to compare Character Recorder with Excel in terms of their usefulness for recording FAIR data and their pedagogical values. Figure 9 shows that participants agree that Character Recorder provides better support for recording FAIR data, with average scores of 6.00 from students and 6.13 from experts. Furthermore, with average scores of 2.75 from students and 2.38 from experts, participants tend to disagree with the notion that Excel is a better tool for learning to document morphological characters.

The matrices created by students and experts were examined, as were the experts’ contributions to the Carex Ontology. A complete student matrix should include 6 characters, 2 samples, and 11-character states (Supplementary Appendix C). Given a character and state pair, “length of stem or culm is 40 cm,” both “length of stem” and “length of culm” were accepted as correct characters in student Excel matrices, whereas in their Character Recorder matrices, only characters using good ontology terms were deemed correct.

Among the Excel matrices created, one did not adhere to the instructions (Participant S211), and two were incomplete (S127 and S213), missing most characters and states. There were 38 errors in total: 9 omitted characters, 27 incorrect character states (including 4 spelling errors of “conspicious” and “lanceloid”), and 2 incorrect character orders. For Character Recorder matrices, one was incomplete (S213), and 28 errors were found, including 19 spelling errors (“conspicious,” “lancoloid,” and “gutter-shape”), 8 incorrect states due to preference for states given in the instruction sheet over recommended ontology terms, and 1 random state. The spelling errors were amplified through multiple reuses of a misspelled value insisted by a participant.

In summary, student Character Recorder matrices were more complete and contained fewer mistakes. Mistakes found in Character Recorder matrices were exclusively in character values and dominated by misspellings. In comparison, 35.7% more mistakes of different nature were found in Excel matrices. Importantly, Excel matrices used 116 distinct words, while Character Recorder matrices contained only 30 unique words, leading to a 74% reduction in vocabulary variation.

### Effectiveness of Character Recorder: expert contribution to the Carex Ontology

**Table 1.** Student task completion time comparison between Character Recorder and Excel

	Min time (s)	Max time (s)	Mean time (s)	Median time (s)	Paired *t*-test
Excel (*N* = 13)	250	1534	515.85	416.0	*P*-value = .03874
Character Recorder (*N* = 13) ^a^	580	1074	783.85	775.0	

a The three students who failed to complete the matrices according to the instructions were excluded.

Experts recorded a total of 1404 complete characters in the time given: 1233 for *C. canescens* and 171 for *C. rostrata*. All experts used the recommended character set, and three of them adjusted by adding new characters or removing existing ones. In total, 43 new characters were created, and 8 of these were utilized by 2 experts. While creating new characters and recording character values, every expert contributed to the Carex Ontology:

1. Sixty-six (66) new terms were added to construct new characters: 22 were landmark terms for numerical measurements, for example, “junction between stem and rhizome,” and 44 referred to organismal structures to be described, such as “stem along a 2 cm segment below inflorescence,” “distal internode of the longest inflorescence,” and “stem base.” New terms were all accompanied by clear definitions.
2. Seventeen (17) new terms were added for character states. Some were good candidate terms such as “scabridulous,” “beneath,” and “overlapping the proximal 2/3 of the perigynia” (to indicate the relative position to another organ). However, 7 terms could be composed using existing terms, for example, “antrorsely scabrous,” “papillose or smooth,” and there were also 2 color terms without definitions (“light yellow-green,” “medium green”).
3. Nine (9) existing ontology terms were enhanced, by receiving a definition, a sample sentence, or being used in a different context. For instance, carex:smooth was initially a subclass of carex:surface_feature, but two experts used the term to describe “texture.” Consequently, the term was put under review for a potential new superclass, carex:texture (carex:toreview, see Fig. 2).

It is important to note that all the new terms added, except for the two-color terms, were accompanied by an informative definition. For example, “primarily pistillate inflorescence units” was defined as “Inflorescence units that are covered by pistillate flowers for more than half of their total length.” This practice would release the definition shortage problem many existing ontologies are facing.

A few issues were identified concerning the form of the terms For instance, some terms began with a preposition (e.g. “in terminal spike”), included conjunctions such as “and/or” within a single term (e.g. “papillose or smooth”), or used plural forms (e.g. “primarily pistillate inflorescence units”). All of these can be avoided through the careful design of error-preventing features in the user interface.

There were three instances where terms with the same meaning were added by different users; for example, “beneath” and “below.” This represents one of the seven types of issues that the Conflict Resolver is designed to handle. The seven types of issues include finding the correct category/superclass, approving definitions, approving new terms, identifying exact synonyms, determining equivalent terms, and resolving disputes over deprecations.

In summary, 92 terms were added or updated by experts during the recording of 1404 characters. The findings suggest that allowing real-time contributions to ontologies by users is an indispensable feature and that experts are capable of providing informative definitions for these terms. Terms contributed in this way would all have an informative definition. Contributions from different users to the same term are logged in one place as annotations, providing rich information that enables ontology managers to incorporate the terms effectively.

### Efficiency of Character Recorder: task completion time

To assess the efficiency of the Character Recorder in recording phenotype characters, we examined participant responses to questions related to the efficiency and task completion times of student participants.

**Figure 10.**
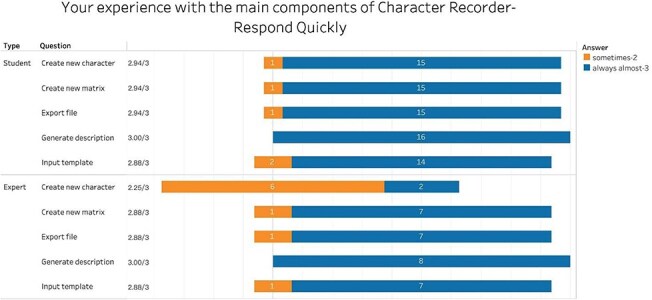
Summary of responses to “Your experience with the main components of Character Recorder - Responded quickly.”

Figure 10 summarizes participants’ evaluations of the efficiency of the five key functions of the Character Recorder. On average, students reported that the components responded quickly, with scores consistently at 2.8 or higher, indicating an “almost always” response level. Experts’ feedback mirrored that of the students, except for *Create New Character*. The disparity can be attributed to experts’ attempts to design novel and intricate characters, such as *the cross-sectional shape of a stem at 90% of the length between the base of the culm to the base of the inflorescence*. These findings suggest that participants perceive Character Recorder as efficient across all functional components except in creating novel characters.

**Figure 11.**
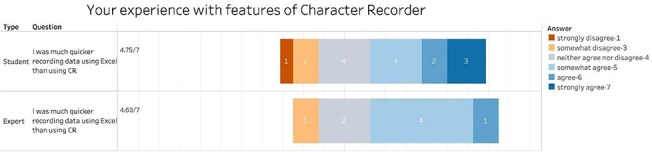
Summary of the responses to “Your experience with features of Character Recorder” in term of task completion speed compared to Excel.

However, students’ task completion time using Character Recorder was significantly longer than using Excel (Table 1, Paired *t*-test, *P*-value = .039). Despite some participants feeling they were quicker with Character Recorder, Fig. 11 shows that with average scores above 4.5, both students and experts feel that Excel was more efficient than Character Recorder.

### Efficiency of Character Recorder: cognitive demands

The user experience questionnaire incorporates a set of widely recognized cognitive load assessment questions from the NASA Task Load Index. Participants were asked to rate their perceived mental demand, performance, effort, and frustration using a 9-point scale. In this study, instead of rating Character Recorder and Excel separately, the scale was presented in comparative terms, where 1 signifies “much weaker than Excel,” 5 represents “the same as Excel,” and 9 indicates “much stronger than Excel.”

The definitions of mental demand, performance, effort, and frustration were included in the questionnaire, and they are:

1. Mental Demand: How much mental and perceptual activity was required (e.g. thinking, deciding, calculating, remembering, looking, searching, etc.) when using CR?
2. Performance: How successful do you think you were in accomplishing the goals of the task (record clear and consistent data) when using CR?
3. Effort: How hard did you have to work to accomplish your level of performance when using CR?
4. Frustration: How insecure, discouraged, irritated, stressed, and annoyed versus secure, gratified, content, relaxed, and complacent did you feel when using CR?

**Figure 12.**
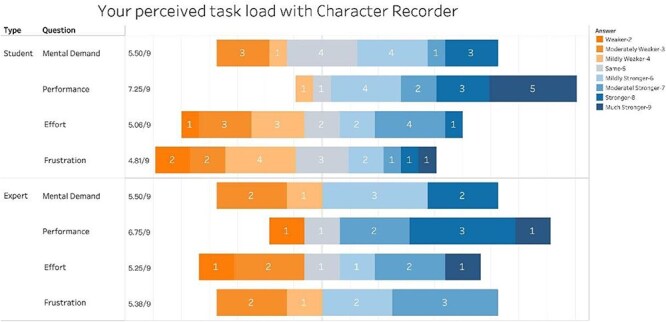
Summary of Participant Responses to “Your perceived task load with Character Recorder.”

Figure 12 summarizes participant responses to these questions. We observed a similar pattern in student and expert responses, with the average scores from student and expert on mental demands, effort, and frustration all close to 5 (between 4.81 and 5.50), while the average scores on performance were 7.25 and 6.75, respectively. These findings suggest that, while Character Recorder imposed a comparable cognitive load in terms of mental demand, effort, and frustration to that of Excel, it offered superior support for recording clear and consistent data. Notably, students perceived this support to be stronger than experts, confirming the finding that students deemed Character Recorder a more effective tool for learning phenotype characters.

In summary, while the average time required for students to complete their assigned task was significantly longer using Character Recorder than using Excel, both students and experts reported comparable mental demands between Character Recorder and Excel, but better data quality using Character Recorder.

### User satisfaction with Character Recorder

The third key aspect of software usability, alongside effectiveness and efficiency, is user satisfaction. To assess the extent to which participants could use Character Recorder to complete tasks satisfactorily, we analyzed participants’ responses to relevant questions in the user experience questionnaire.

**Figure 13.**
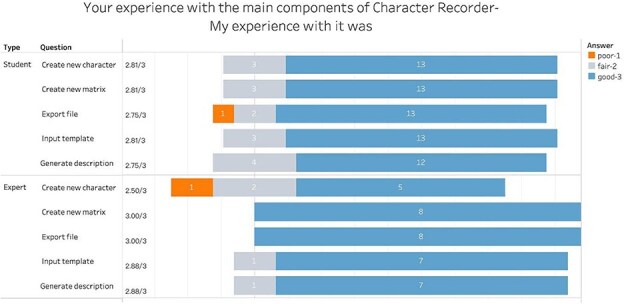
Summary of responses to “Your experience with the main components of Character Recorder: My experience with it was.”

Figure 13 summarizes users’ experiences with the five key components of Character Recorder. The average scores from both student and expert participants ranged from 2.75 to 3 out of 3 for all components except for *Create New Character*. This indicates that participants had good experiences with the other four components, but enhanced support for creating novel characters is needed. This finding aligns with previous observations regarding the software’s effectiveness and efficiency.

We further examined participants’ reported experience with various aspects of the Character Recorder. Figure 14 summarizes their responses to the set of questions.

**Figure 14.**
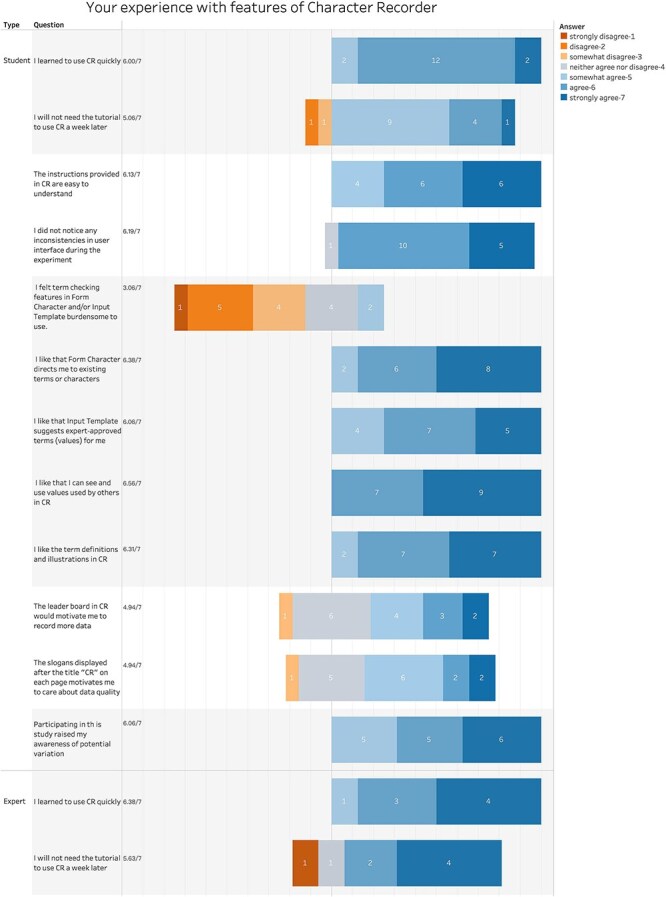
Summary of participant responses to “Your experience with features of Character Recorder.”

**Figure 14.**
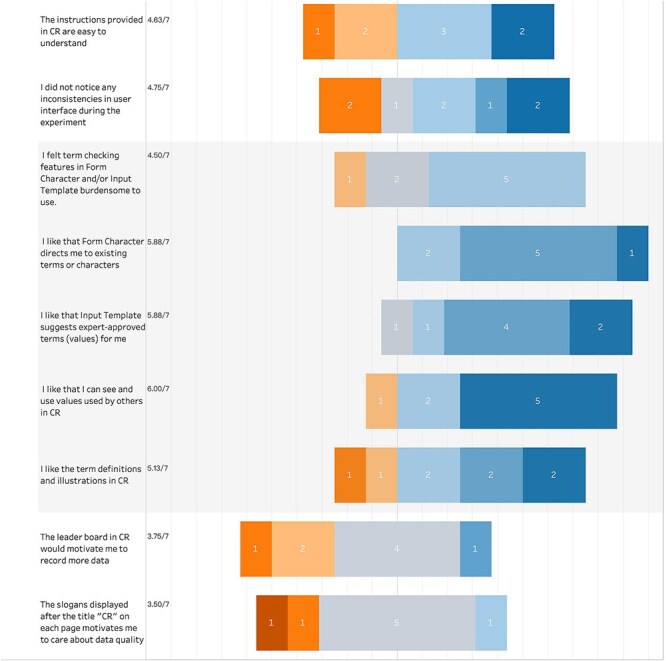
(Continued)

On the learnability of the software, both students and experts reported that they learned to use the Character Recorder quickly (student score = 6.0; expert score = 6.38) and believe they can likely pick it up in the future without needing a tutorial (student score = 5.06; expert score = 5.63).

On the consistency and instructional clarity of the software, student scores were noticeably higher than expert scores. Specifically, students rated consistency at 6.19 and instructional clarity at 6.13, where 6 indicates “consistent and clear,” while experts rated them at 4.75 and 4.63, respectively, where 4 signifies “neutral” and 5 signifies “somewhat consistent and clear.”

On the ontology-supported and value reuse features, students expressed agreement with all the statements in the four “I like..” questions, with average scores exceeding 6.00. This indicates that students found the definitions and illustrations provided by the software helpful, aligning with previous findings on the utility of character illustrations [16]. Additionally, students valued the guidance offered by Character Recorder, which leverages the Carex Ontology for character formation and value selection. Despite the introduction of extra steps, such as consulting the Carex Ontology and validating user entries, students viewed these steps as manageable rather than burdensome. This is reflected in the average score of 3.06, which suggests students somewhat disagreed with the notion that these steps were burdensome.

In contrast, experts gave an average score of 4.50, a stance between “neutral” (4) and “somewhat agree” (5) on that question, suggesting they found these additional steps more burdensome than students did. Furthermore, although experts also found the definitions and illustrations helpful, their average score of 5.13 was noticeably lower than the students’ score of 6.31. These differences between students and experts may stem from the experts’ extensive knowledge of *Carex* and the complexity of their tasks, which require deeper engagement with the Ontology.

Like the students, experts appreciated the guidance provided by the software on character formation and value selection, as evidenced by the scores of 5.88. They also valued the ability to see and use values entered by others, with a score of 6.00. However, one expert voiced a concern during the hands-on session that this feature might impact an expert’s independent assessment of a character.

One question, specifically tailored for students, inquired whether their participation in the study heightened their awareness of the variations in character expressions. With an average score of 6.06, the students’ responses confirmed that (Fig. 14).

### Color palettes

We employed data mining techniques and created a set of color palettes from a color dataset. This color dataset was meticulously compiled by measuring the sRGB values of colors from high-quality images of *Carex* specimens with color calibration. The dataset contained 2883 colors of various organs and provided a sufficient number of observations to derive color palettes for brown, green, red, yellow–brown, and yellow–green. Details on the creation of the color palettes can be found in Reference [18]. In Character Recorder (Fig. 5 a.2), experts were free to pick a color from these color palettes or enter textual color names for color-based characters.

**Figure 15.**
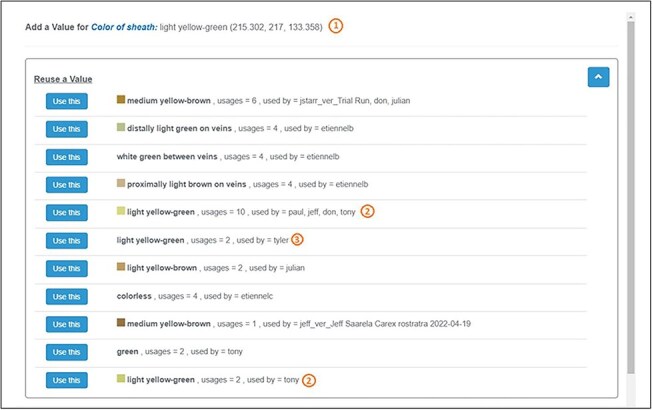
Expert recorded colors for the color of sheath of *C. canescens*.

In the experiment, experts documented a total of 78 color-based characters. Out of these, 21 were not represented in the provided color palettes, for example, “white,” “colorless,” or “blue-green.” Of the remaining 57 colors that were included in the palettes, 9 were recorded as textual color names and 48 used the palette colors, amounting to 84% usage of the palettes. This high utilization rate suggests that the color palettes were generally suitable for the task. Although finding an exact color match to what experts observed can sometimes pose a challenge, the results underscore the benefit of recording colors to enhance the FAIRness and computability of character descriptions. As illustrated in Fig. 15: (i) Character Recorder captures both sRGB values and a textual name when a user selects a color from a palette. (ii) Recording colors as sRGB values allows for the computational measurement of differences between colors labeled the same, enhancing data usability and interoperability. (iii) In contrast, when colors are recorded as plain text, they lack reliability for comparison, diminishing their usability, reusability, and interoperability.

### Overall comparison and user recommendation

**Figure 16.**
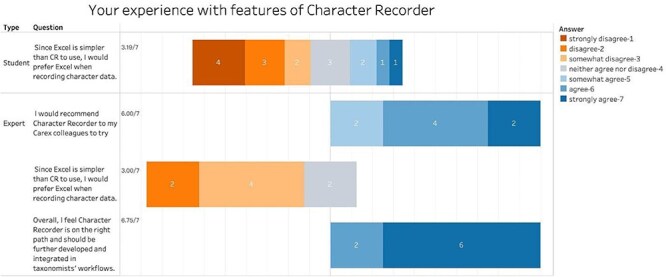
Summary of participant final comparison between Character Recorder and Excel and their recommendations.

Figure 16 summarizes participant responses regarding their preferred choice for recording FAIR data, alongside experts’ recommendations. The results indicate that both students and experts lean toward disagreeing with the notion that Excel would be their tool of choice, with average scores of 3.19 and 3.09, respectively (3 means “somewhat disagree”). Additionally, experts would recommend Character Recorder to their colleagues (average score = 6.0). Furthermore, there is a strong consensus among experts (average score = 6.75 out of 7) that Character Recorder is moving in the right direction and should be further developed and integrated into taxonomists’ workflows.

## Discussion

### Potential for Character Recorder or the like to replace Excel as the matrix editor

Despite the development of several character matrix editing software applications over the years, Excel remains the most popular, largely due to its versatility and lack of constraints on user input. Comparing Character Recorder with Excel allows us to determine whether Character Recorder, with its necessary ontological constraints for FAIR data, is a viable replacement. A significant focus in designing Character Recorder was on integrating ontology-supported features in a user-friendly manner to mitigate potential cognitive burdens. The findings indicate that participants perceived similar cognitive demands between Character Recorder and Excel while recognizing the superior quality of data recorded using Character Recorder. This is encouraging.

One key finding from the 2022 survey on the attitudes of phenotype data producers and consumers revealed that while respondents preferred author curation over third-party curation for published character descriptions, 50% of participants believed biologists lack of skills to curate their own characters using ontologies while only 22% disagreed [13]. The findings reported here provide evidence that it is possible to develop user-friendly editing platforms suitable for biologists across all experience levels to make their data FAIR. Experts contributed good terms and informative definitions to the Carex Ontology while filling out their matrices. A vast majority of participants reported that they quickly learned how to use Character Recorder. All ontology-supported features, and the character share and reuse features, were well-received by both undergraduate students and professional *Carex* systematists.

### Improvements to Character Recorder

**Figure 17.**
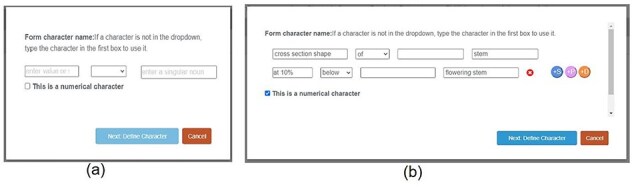
A before (a)-vs-after (b) comparison on the Form Character window.

As a proof-of-concept prototype, Character Recorder has areas in need of improvement. Specifically, the creation of novel characters and the selection of recommended characters were identified by experts as requiring enhancements. The prototype lacks flexibility in supporting novel characters and it is also limited in its capability of intelligently recommending characters for various taxa. We actively engaged with experts to discuss potential improvements and implemented those after the Experiments. Figures 16 and 17 provide a before-and-after comparison, showcasing the enhancements made to the “Form Character” window and the interface for recommended characters, respectively.

Figure 17 (a) shows the Form Character window used in the experiments. Before values are entered (e.g. “pubescence” “of” “leaf”), the text boxes display brief instructions. Figure 17 (b) illustrates how the enhanced character window enables users to construct complex characters, incorporating an unlimited mixture of structural constraints (+S, associated with some organ), positional constraints (+P, indicating the location for measurements, e.g. at mid-height), and distance constraints (+D, relative to a reference organ). Shown here the “cross section shape of stem” is constrained with a Distance constraint “at 10% belowig flowering stem”. In future development iterations, rather than requiring users to manually complete fields, the system could employ a natural language parser to translate users’ natural language descriptions into structured representations.

**Figure 18.**
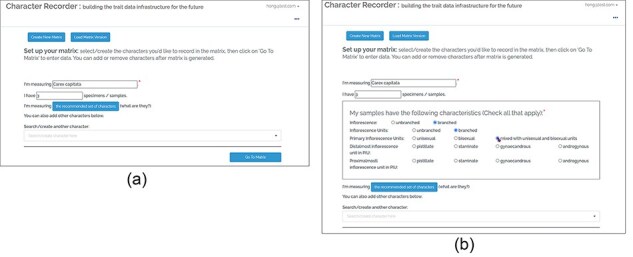
A before (a)-vs-after (b) view on the recommended characters.

Figure 18 (a) shows the user interface used in the experiments. All taxa have the same set of core characters recommended for users to record. The Improved version shown in (b) incorporates a hand-crafted decision tree, which allows Character Recorder to recommend specific core characters based on the taxon in question. The selections users make at each step determine the subsequent questions and options displayed. For instance, if the taxon’s inflorescence is unbranched, questions related to inflorescence units will not appear. Although not depicted in the figure, the “recommended characters” list dynamically adjusts according to the user’s responses. This decision tree will likely be taxon-specific for different taxonomic groups.

Other features experts discussed included color, surface feature, and internal textual terms and their groupings in the Carex Ontology. Experts reached a consensus of creating new classes carex:margin_shape, carex:apex_shape, carex:vestiture (as superclass for surface features and pubescence), and carex:internal_textual in the Ontology.

The feature for sharing and reusing character values was favored by all student participants, largely attributable to its potential to improve productivity. However, an analysis of matrices produced by students revealed that two misspelled character values (“conspicious” and “gutter-shape”) were introduced and then reused by many others, leading to a total of 18 instances of misspelled values. In contrast, during the Expert Experiment, we noted that experts reviewed values recorded by others and alerted one another about questionable entries. In examining the matrices produced by the experts, no cases of misspelled values were observed. The insight we discerned from these findings and observations is that the feature for sharing and reusing character values is not only beneficial for enhancing productivity and reducing variability but also serves as a means of peer quality control at the expert level. Nonetheless, the integration of a spelling checker would be necessary for novices to mitigate the risk of misspellings.

### Next steps and code sharing

Being a proof-of-concept system, Character Recorder and other components of the platform are not fully developed nor is it evaluated at a larger scale. After confirming our work has shed lights on ontology design tips and helpful features, following the hands-on session, experts suggested that the next step in the development of this ontology-enhanced data production platform would be to invite more fauna and flora communities to further develop and evaluate it under different context and with more diverse use cases. Areas needing attention include, but are not limited to, the taxon-specific recommended character sets and associated decision tree (see Fig. 18), formulation and full ontologization of novel characters, comprehensive test of Conflict Resolver with all seven types and new types of ontological issues, and intellectual rights issues with character sharing.

In this article, we described the creation of the Carex Ontology in detail, which may be an accessible way for other taxon groups to create their first ontology. These domain-specific ontologies are expected to work under their reference ontologies. For plants, the reference ontology is the Plant Ontology. Carex Ontology and all the source code of the platform are available under CC0 and accessible via the Readme page of https://github.com/biosemantics/character_recorder.

### Study design rationale and limitation

We conducted experiments with both undergraduate biology students and *Carex* systematists at varying career stages for several reasons: (i) To instill an early awareness of data quality issues among students. This objective was met, as students reported an increased awareness of data variation following the experiment. (ii) To gather insights from a wide range of potential users of a tool like Character Recorder, thereby informing future research and development. This goal was also achieved, as the findings reveal both a consensus between the groups and some notable differences. (iii) For the Expert Experiment, to assess the chance for experts to adopt Character Recorder or the like in their professional work we must provide a life-like setting for experts to experience Character Recorder in the hands-on session, risking potential biases that may be introduced through the peer interaction. To avoid introducing biases, the request for participants to provide their honest assessment was made both verbally during the session and in writing in the questionnaire. The facilitator encouraged peer discussions during the hands-on session and was open to all questions, comments, and critiques. The questionnaire was filled out by participants independently and anonymously in the end. Through those measures, we tried to make sure the responses to the questionnaire represent participants’ *informed* (through peer discussions) and *true* (through the pressure-free environment and anonymity) opinions. The (biology) Student Experiment, on the other hand, was well-structured and student participants completed their sessions without interacting with other participants. The findings of the two experiments are quite consistent, with differences well explained. However, we acknowledge the limitations posed by the small participant size in the Expert Experiment, which precluded statistical analysis of the findings. Nonetheless, the results are valuable and can inform the development of similar software applications.

## Conclusion

In this article, we described an author-driven FAIR data production platform and the evaluation of its key component, Character Recorder, a proof-of-concept prototype of an ontology-enhanced taxon-by-character matrix editing application.

The findings from the two evaluative experiments with biology students and *Carex* experts consistently confirm that ontology-supported FAIR data features are liked and usable by both groups of participants, adding evidence that authors are capable of producing computable data at the time of publication of their phenotypic data using ontologies. Character Recorder incurred a cognitive load comparable to that of Excel, yet it allowed participants to produce data that are more FAIR than Excel: all data points produced with Character Recorder were associated with ontological definitions; landmarks involved in all numerical measurements were explicitly defined; 84% of color values covered by the color palettes were associated with their sRGB values; and the number of unique words used in a matrix was dramatically reduced thanks to the guidance the Carex Ontology provided.

After using Character Recorder for 3 days, all expert participants agreed that the tool was on the right track and should be further developed and integrated into taxonomists’ workflows. Despite significant advances in Natural Language Processing through large generative language models like GPT, these technologies cannot outperform post-doctoral data curators in resolving ambiguous characters. It is essential to equip authors with practical tools to formalize the meanings of their characters. While making the Carex Ontology and all software source code CC0, we encourage further evaluation of the Character Recorder methodology under different contexts and with different use cases.

Based on the findings reported here, we propose that both existing and future tools to consider support these useful features. For the backend ontologies, include the specific semantic constructs we have included in the Carex Ontology that support: (i) system recommend characters to ensure parallel descriptions; (ii) recommended and not recommended character and character state terms to minimize variations and encourage terminology convergence; (iii) users add terms and definitions to facilitate efficient workflow and to encourage ontology adoption; and (iv) illustrations of character, character states for enhanced user experience. For user interface, consider support: (i) notifications about ontology updates to integrate ontologies seamlessly into user workflows; and (ii) share and reuse character and character states for peer-based quality control, variation reduction, and improved productivity; and (iii) domain-specific color palettes to support computable color feature.

## Supplementary Material

baae097_Supp

## Data Availability

The material used in student and expert studies is available in the supporting information of this article as supplementary data. The source code of the software and the Carex Ontology are publicly accessible on GitHub at https://github.com/biosemantics/character_recorder.
