# Supplementary material for: Helping authors produce FAIR taxonomic data: evaluation of an author-driven phenotype data production prototype
Source: Database (Oxford). 2025 Jan 29;2025:baae097. doi: 10.1093/database/baae097 (PMC11928229; doi:10.1093/database/baae097)
Supplement: baae097_Supp [file baae097_supp.zip › supplementary file.docx]

Appendix A

Demographic Questionnaire _ Authors in the driver's seat: fast, consistent, computable phenotype data and ontology production.

Your ID: ___ (Each subject will be given an 3-digit ID.)

Age: What is your age range

20-24

25-29

30-39

40-49

50-59

60-69

>69

Gender: What is your gender?

Male

Female

Transgender

Other

Prefer not to respond

Are you ___?

A Master student

A Ph.D. student

A Post-Doc researcher

Completed all the formal trainings in botany [e.g., faculty member, collection manager, research scientists]

How would you describe your knowledge in morphological characters of *Carex*? [select the greatest number that applies to you]

1. I know little about morphological characters of *Carex*
2. I am/was exposed to those in an undergraduate course
3. I am/was exposed to those in a graduate course
4. I acquired such knowledge through informal learning
5. I teach/taught those to college students
6. I authored taxonomic descriptions for *Carex*

**Appendix B:**

**Character Recorder Tutorial**

**[Please carefully follow this tutorial to learn to use Character Recorder. Read the text and not just the graphs. You may keep this page open in a browser tab in case you need it during the experiment]**

In this tutorial, you will learn to use Character Recorder to enter the following information for a Carex taxon.

| The Carex Taxon**: *Carex* arctata**  You have**: 2 samples of *Carex* arctata**  Record the following characters for the taxon:  **Sample 1:**  Width of leaf blade = 3 mm  Color of leaf margins (i.e., leaf blade margins) = rusty (i.e., ferruginous)  **Sample 2**: `  Width of leaf blade = 1 cm;  Color of leaf margins (i.e., leaf blade margins) = red |
| --- |

| **The general steps of using Character Recorder to create and export a character matrix:**   1. **Enter taxon name and number of samples** 2. **Select or create *all* characters needed to describe *every* sample** 3. **Generate the matrix and enter values in the matrix** 4. **Throughout the process, take the suggestions made by the software and reuse existing characters and values whenever possible.** 5. **Generate one textual description per taxon and export the matrix and description files.** |
| --- |

| **Registration and login** |
| --- |

1. You should have received a Participant ID (PID) from the researcher.

2. Go to <http://shark.sbs.arizona.edu/chrecorder/public/login>

**
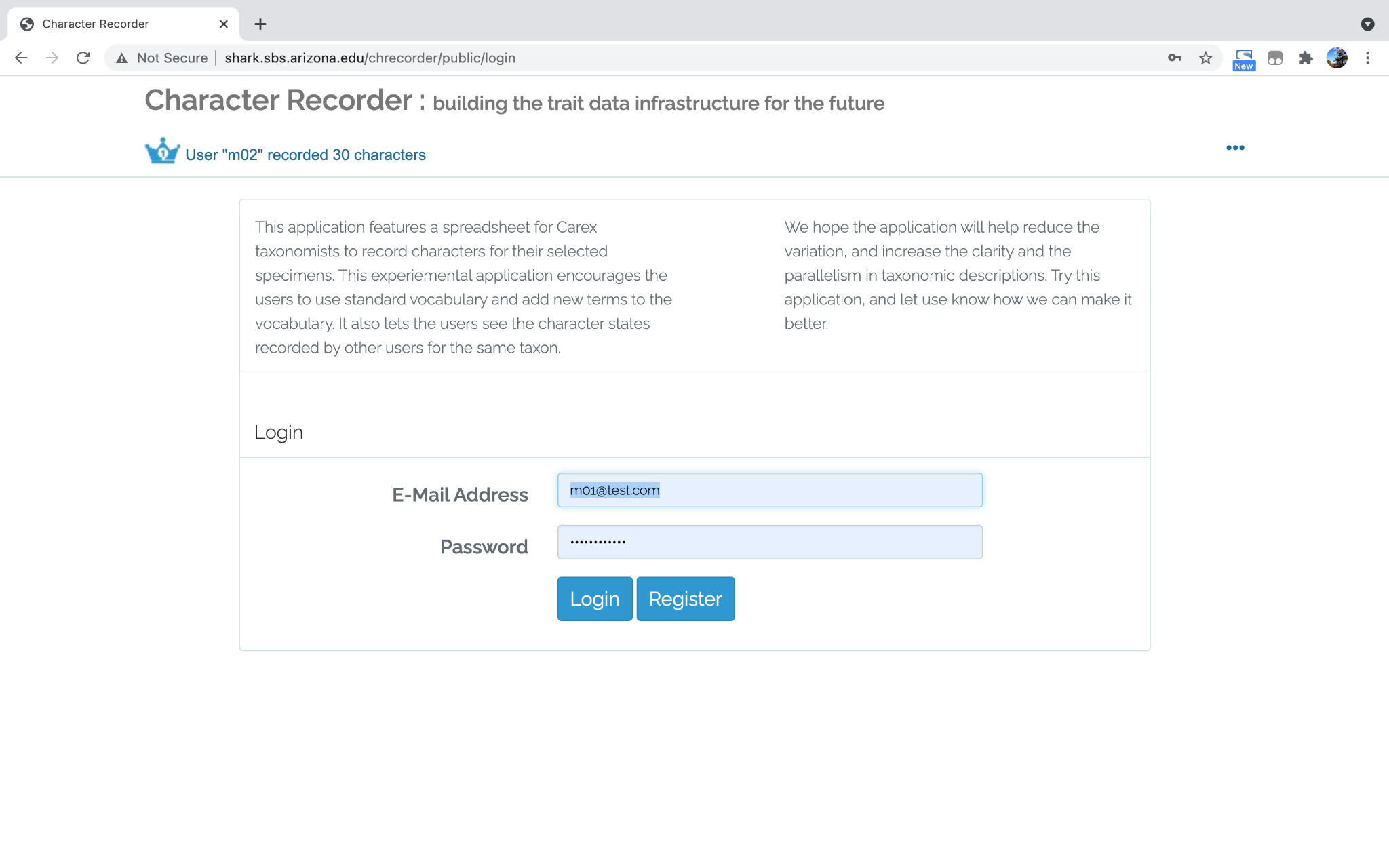
**

- Read the introduction text
- **Register** an account using the following information:

Email address: [*your_PID]*@test.com. (Use your own PID to replace [*your_PID*], for example: s111@test.com)

Password: any 6-character-long string is good for the password.

Note down this password as you will use it again.

| **Select/create characters: take a look at “Recommended Characters”** |
| --- |

3. After registration, you will be directed to the following page:

**
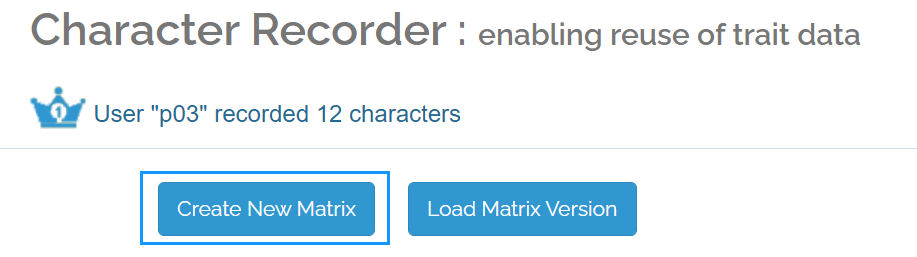
**

- Click “Create New Matrix”

4. Now you are on the “Set up your matrix” page. **To set up a matrix for a taxon, you need to select *all* characters you want to describe. If the characters you need do not exist, you need to create those.** You could choose
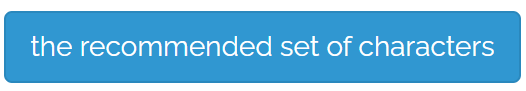
, which is a set of 100 characters Carex experts recommend. But since in this tutorial you only need to record a few characters, selecting or creating the needed characters directly is more effective than selecting the needed characters from this long list of recommended characters.


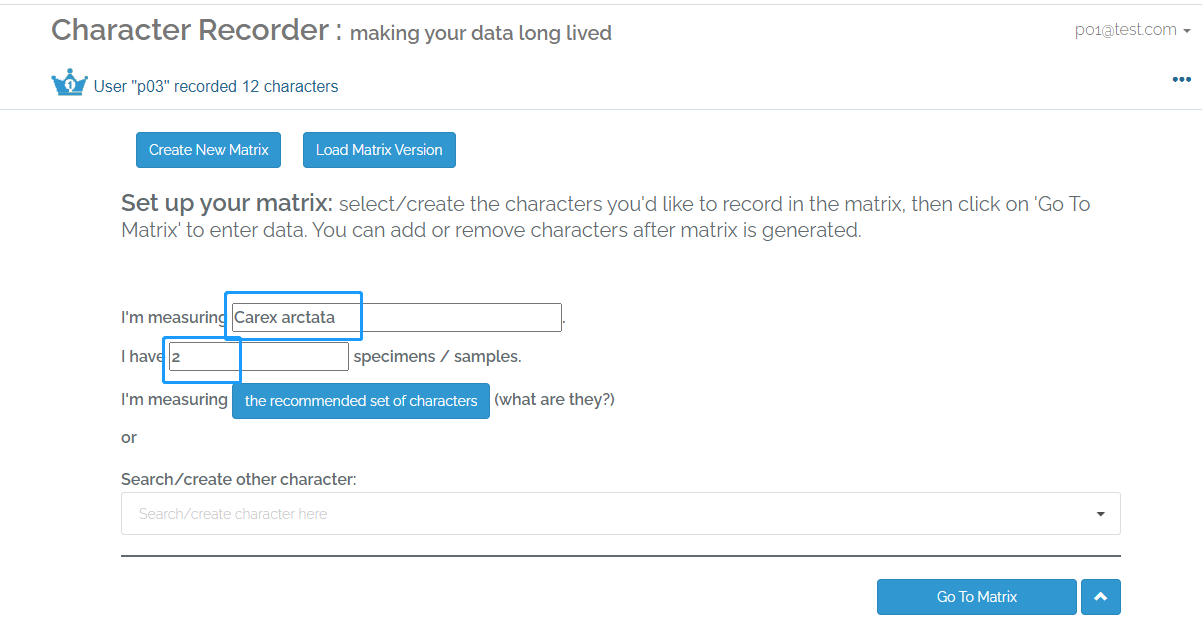


- Input taxon name **Carex arctata**
- Input number of samples to describe, 2.
- Click
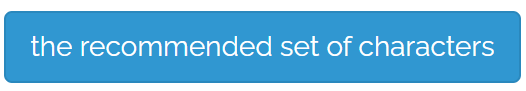
 and see the characters being listed at the lower portion of the page. Quickly scan through the list. Since you are not going to use most of these characters, use the top “x” to remove the entire recommended character list (see the image below).


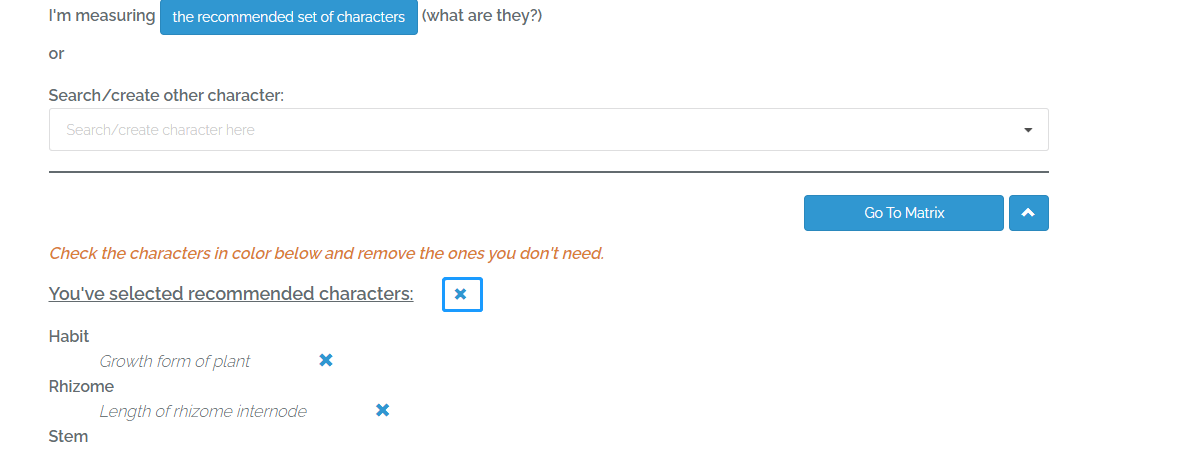


| **Select/create character: select the 1st character “width of leaf blade”** |
| --- |

5. First search and see if ‘width of leaf blade’ exists. In the “Search/create other character” box,


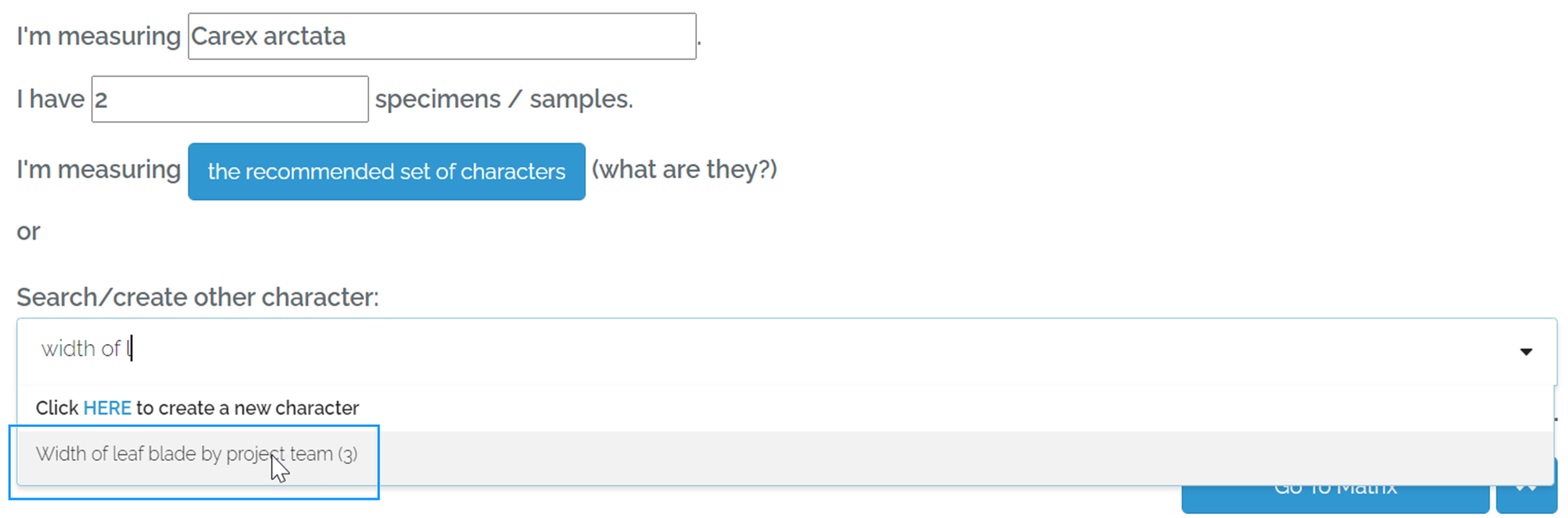


- Enter “leaf blade” or “width of leaf blade” to search. The search box will display all characters containing the input string. Find “width of leaf blade” in the filtered list and select this as the first character.
- In the “Define character” window:


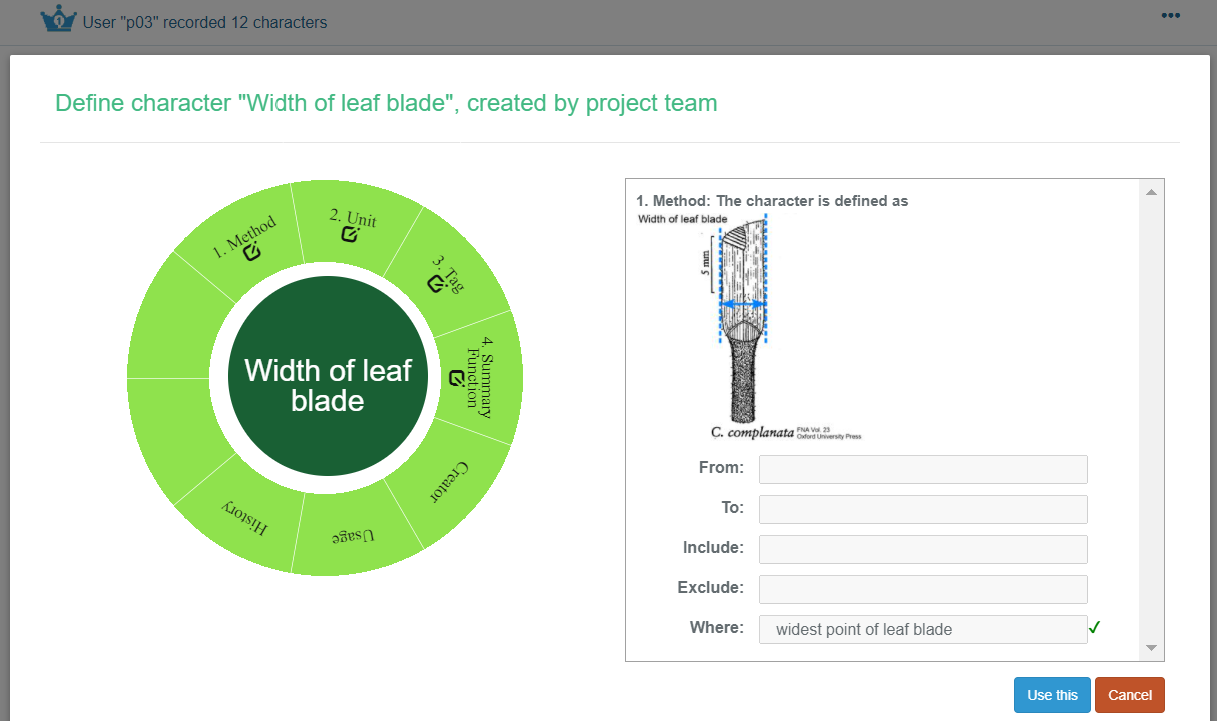


- Review the Method, Unit, Tag, and Summary Function sections of the character definition by clicking on the relevant segment in the green wheel. A numerical character such as width, length, or diameter has information in the Method and Unit sections, while a categorical character such as color or shape only has information in other sections. For example, clicking on Tag will reveal how tags are used:


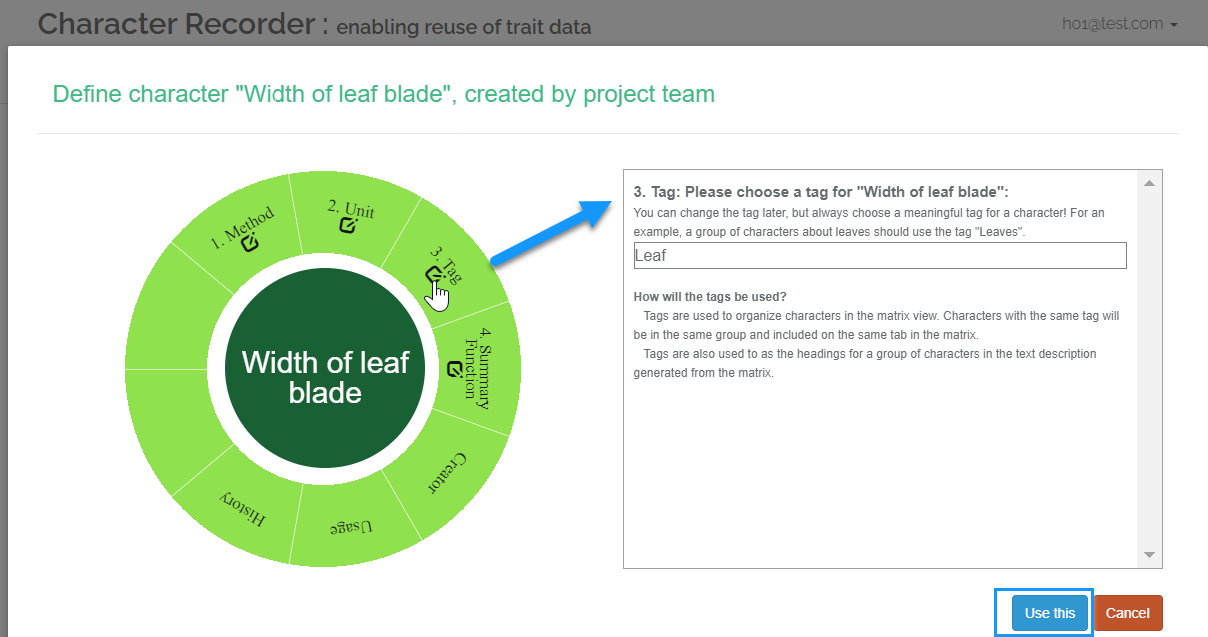


- The information reviewed suggests that this is the character you want. Click
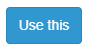
 to save this character in your character list. You may need to zoom out in the browser to make
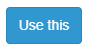
 visible.
- You should see that “width of leaf blade” is listed now in your selected character list (see the image below). Yes, “width of leaf blade” is one of the 100 recommended characters.


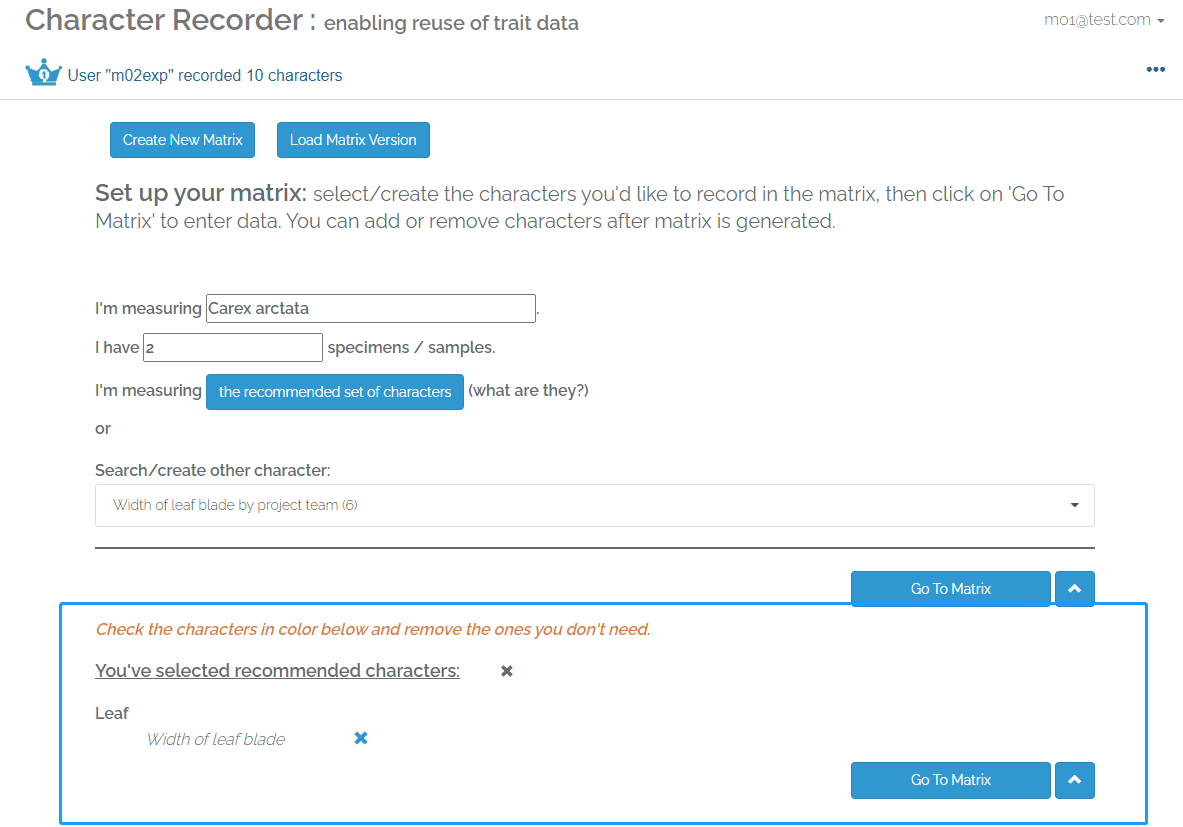


| **Select/create character: create/select the 2nd character “color of leaf margins ”** |
| --- |

6. Now, search and see if ‘color of leaf margins’ exists. In the “Search/create other character” box:

- Enter “leaf margins” or “color of” to see if there are any matches. If a match is found, then select the character, and
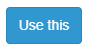
 just like you did for the 1st character. Then skip the rest of the steps in this section and jump to the [Go to matrix] secion.
- If no match is found for “color of leaf margins”, you need to create the character anew, using the “Search/create other characters” box:


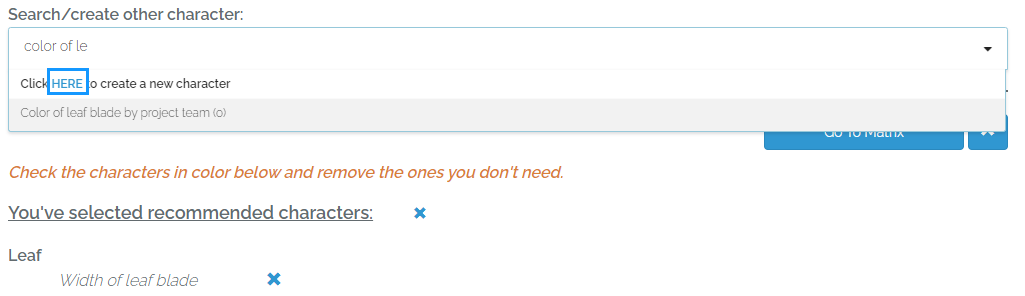


- Click in the ‘Search/create other character’ box to invoke the dropdown list. Click
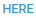
 in the first entry of the list (see image above) to start creating a new character procedure.


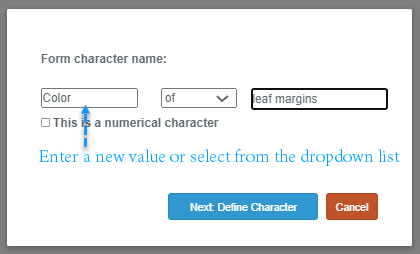


- Form the name for the character as shown above. **Note: All dropdown selections in Character Recorder also accept user input. So you can enter a new character in the first box above if the dropdown list does not contain what you need**.
- If this character were a numerical character (like length, width, or diameter that takes numbers as its value), you would need to check the “**This is a numerical character**” checkbox. Since color is a categorical character, you will leave the checkbox unchecked.
- Next, click
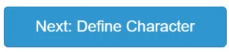
.


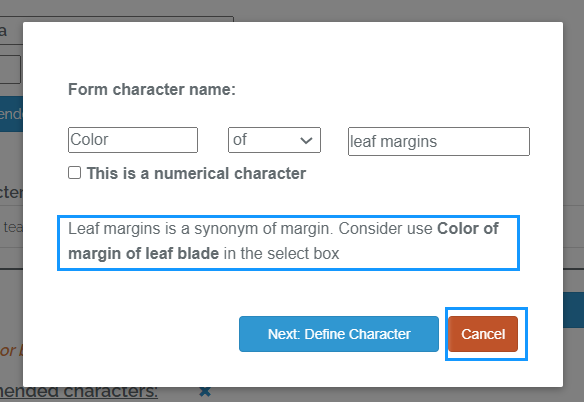


- The software informs you that “Color of margin of leaf leaf blade” exists, which is just a different way of saying “color of leaf margins”. Click on
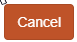
 and go back to the Search/create character box, and select and use that character.


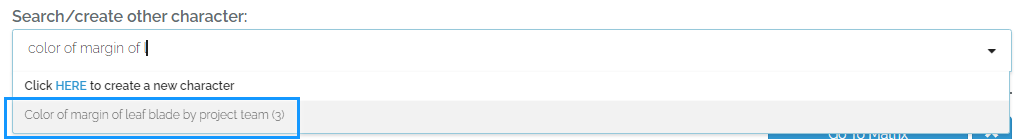


- If the software suggests other things, please follow the software’s suggestions.

| **Go to matrix** |
| --- |

7. You are now back to the “Set up you matrix” page. At this time you have selected/created all two characters needed for your matrix: both of them are actually in the recommended character set!


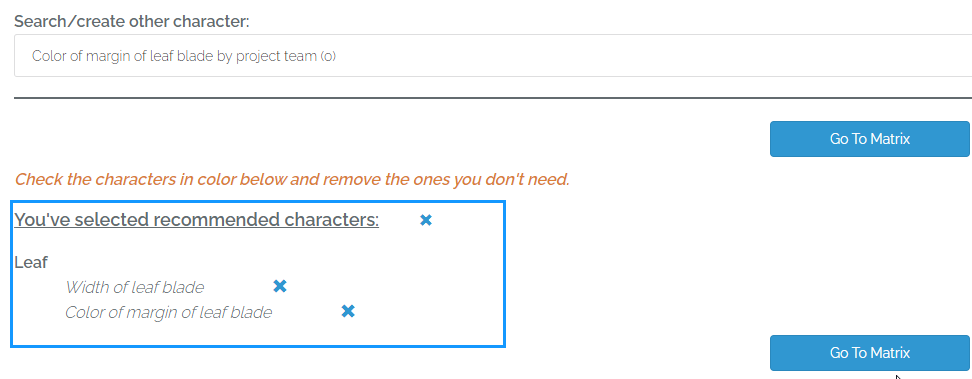


- Click
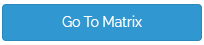
 to land on the matrix page (shown below).


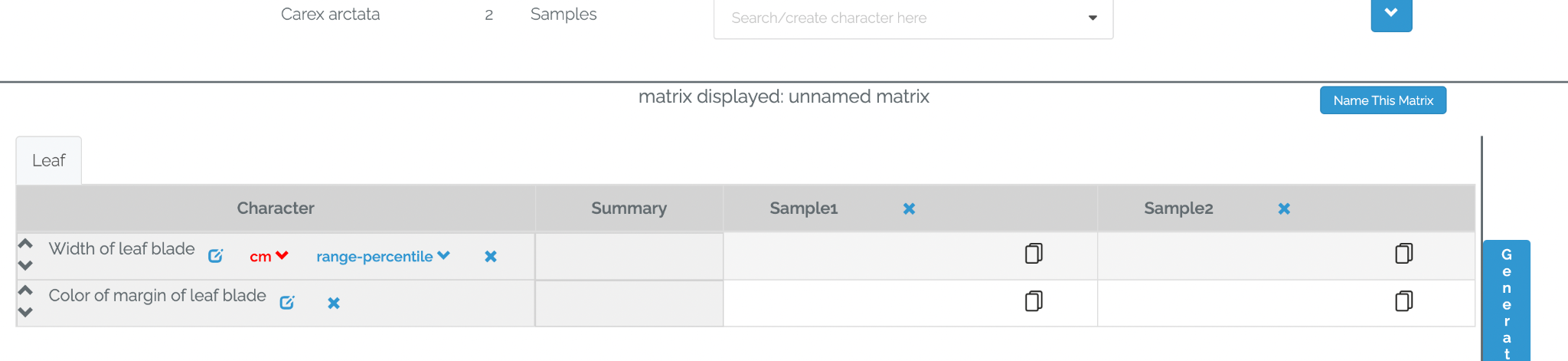


| **Input values into the new matrix** |
| --- |

8. While the matrix is displayed, enter values for the two characters:


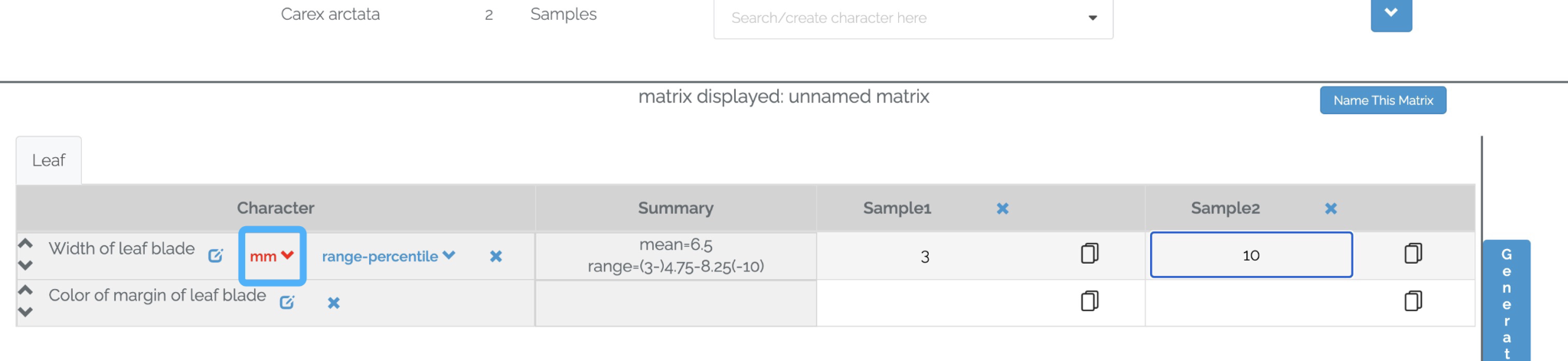


- On the row of “width of leaf blade”, enter the value as shown in the image above. Remember to click on the unit to select one unit consistently for all the samples, e.g., “mm”, The software finds the minimal and maximal values entered in the row and the range in which the most values fall. It then presents the result in the format of (min-)common_range(-max) in the Summary column.
- On the row of “color of margin of leaf blade”, click in the data cell of Sample1 to invoke the “Add a value” input template to enter a color value.


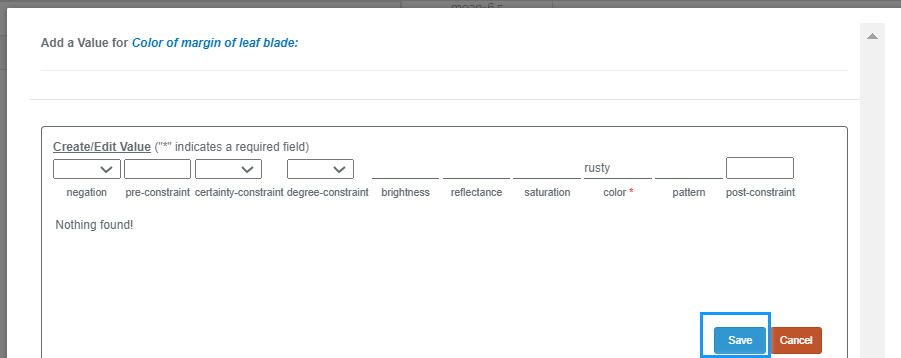


- - Enter “rusty” on the ‘color’ line and click
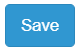


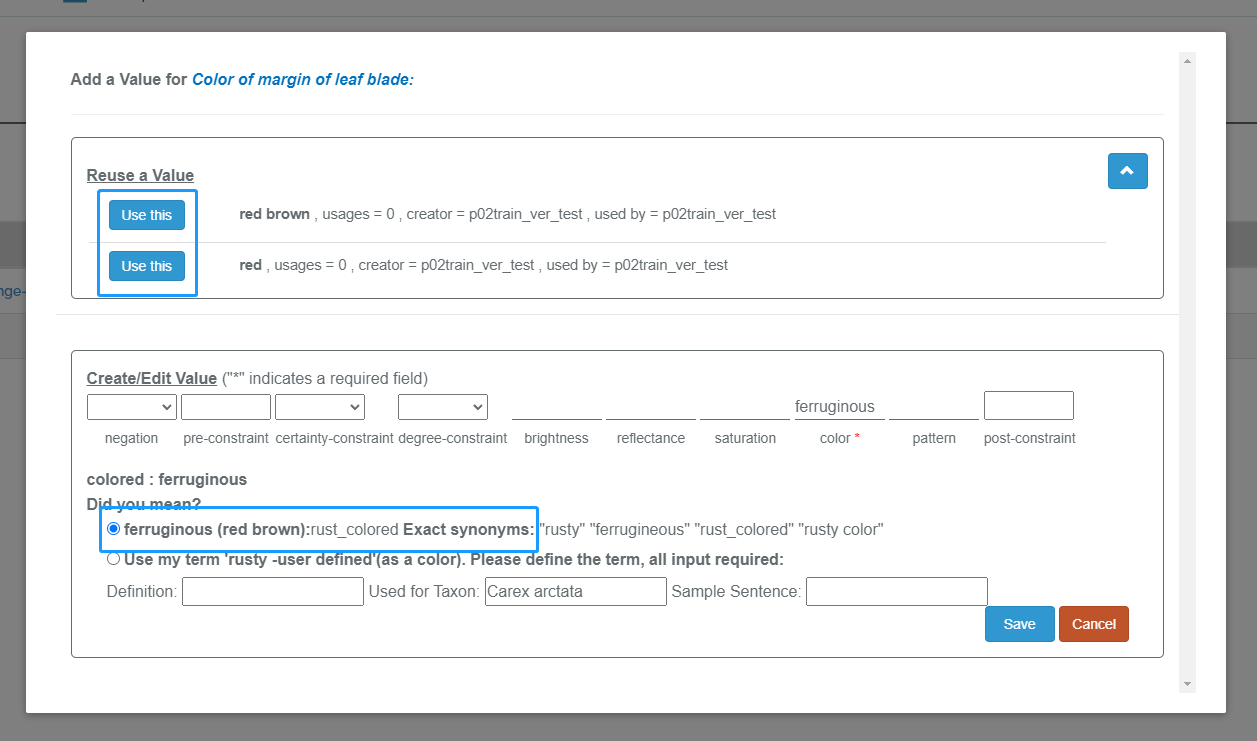


- - Observe that the software knows that “ferruginous” means “rusty”, so check the radio button by ferruginous and click on
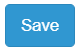
. This way, all ‘rusty”, “rust colored” etc. will be standardized to ferruginous.
  - Note: other users have used “red”, and “red brown” in the past for this character of this taxon. If any of these values is what you need, click on
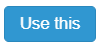
to reuse the value. Reusing existing values reduces variations and improves consistency!
  - For Sample2, enter or select ‘red’. See image below.


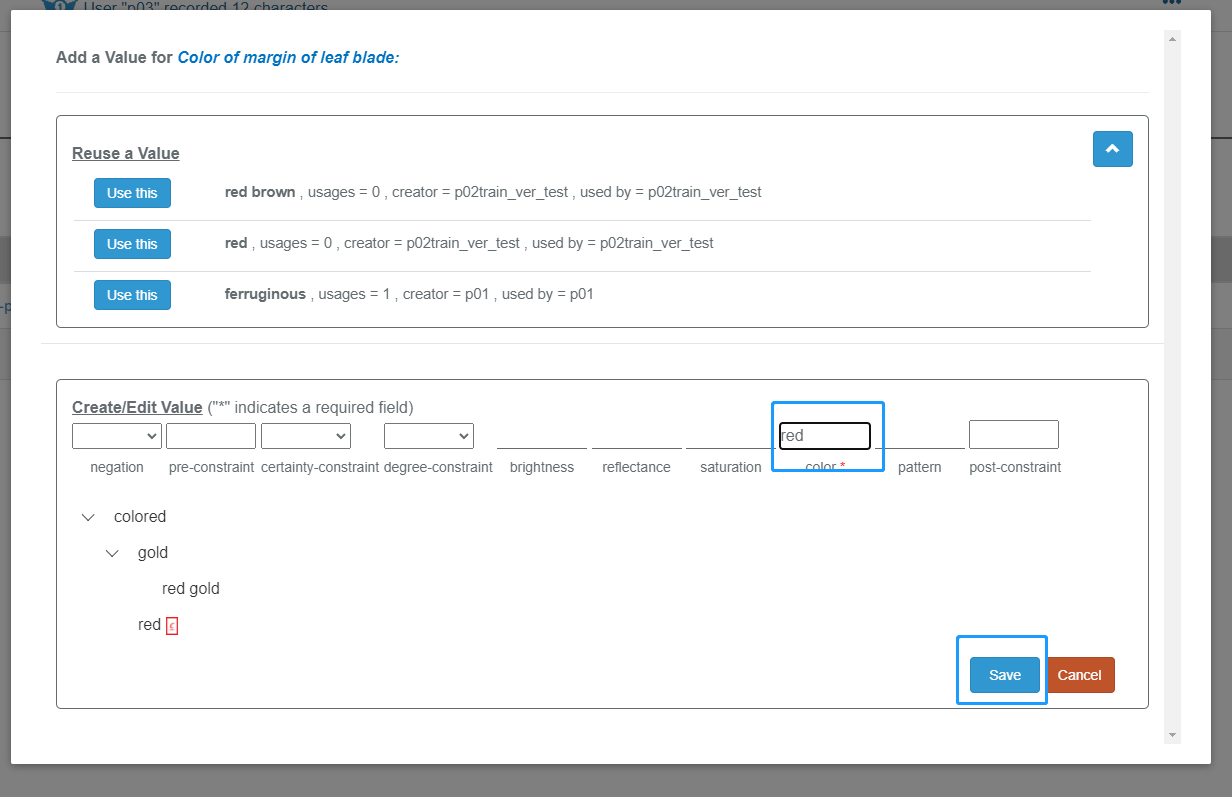


- At this time, the content of your matrix should look like what is shown below. Check that you have the correct taxon name, number of samples, two characters, and appropriate values.

Carex arctata 2 samples

| Character | Summary | Sample1 | Sample2 |
| --- | --- | --- | --- |
| Width of leaf blade (**mm**) | Mean = 6.5  Range = (3-)4.75-8.25(-10) | 3 | 10 |
| Color of margin of leaf blade |  | ferruginous | red |

| **Name/save your matrix** |
| --- |

9. Click
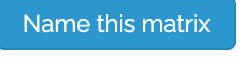
 to give your matrix a name “test”. The matrix name will then be displayed (see image below). You can also access this matrix in the future through the
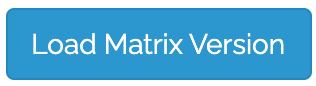
button.


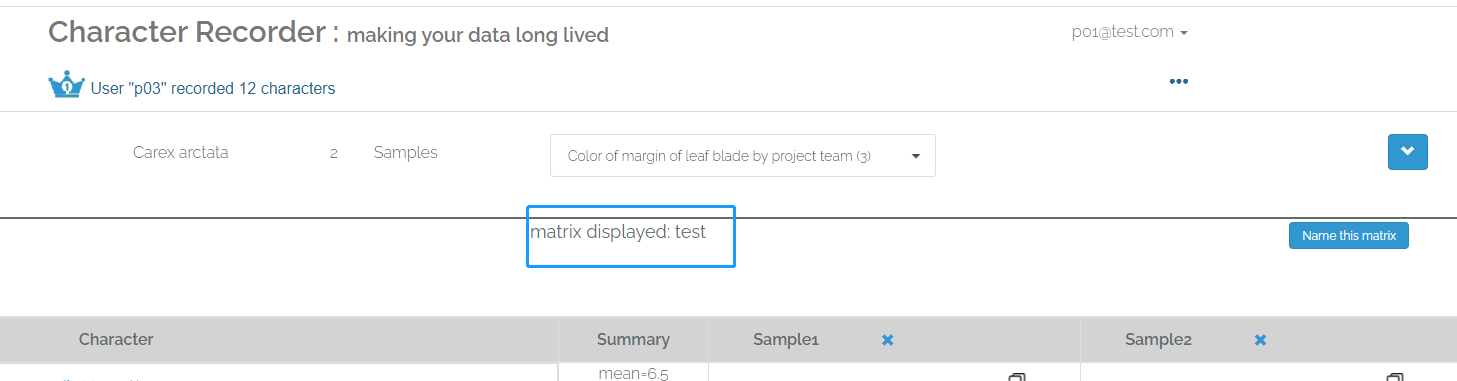


| **Generate textual description and export files** |
| --- |

10. Academic journals often require both matrix and narrative forms of character information. On the matrix view, you can generate a narrative description of the characters you just entered and export both the matrix and narrative as a csv/trig file and a doc file.


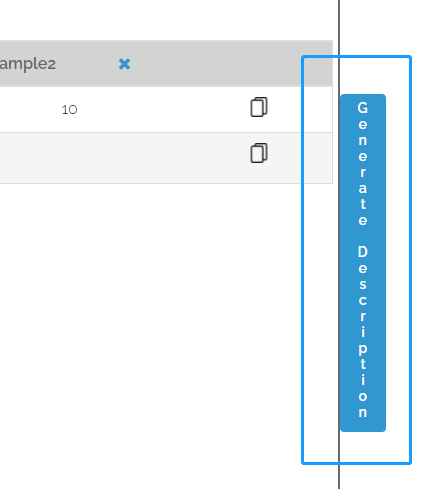


- Click “Generate description”, and read the description the software generates.


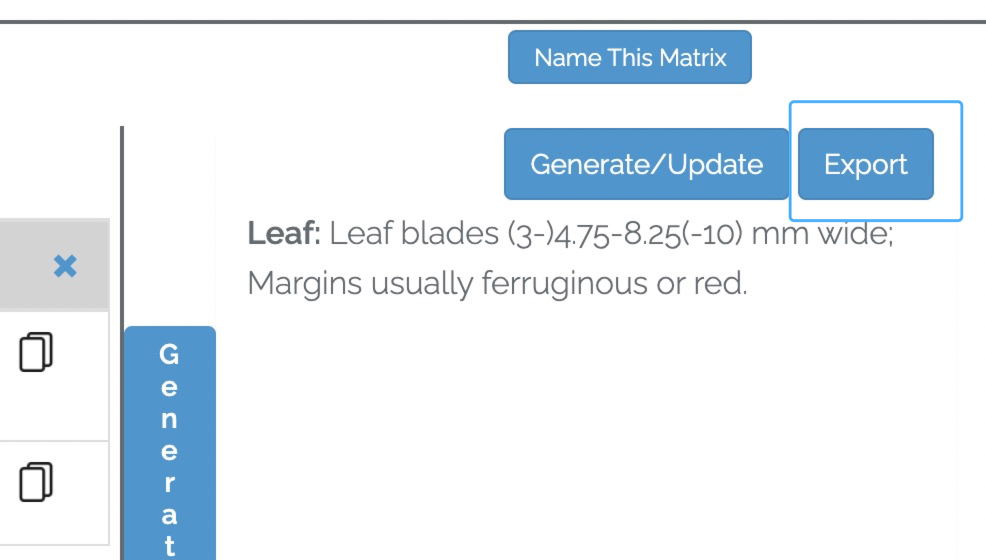


- Click
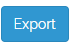
. A zip file is downloaded holding three files (e.g., “Carex actata.csv, .docx, and .trig'') to your “downloads” folder.


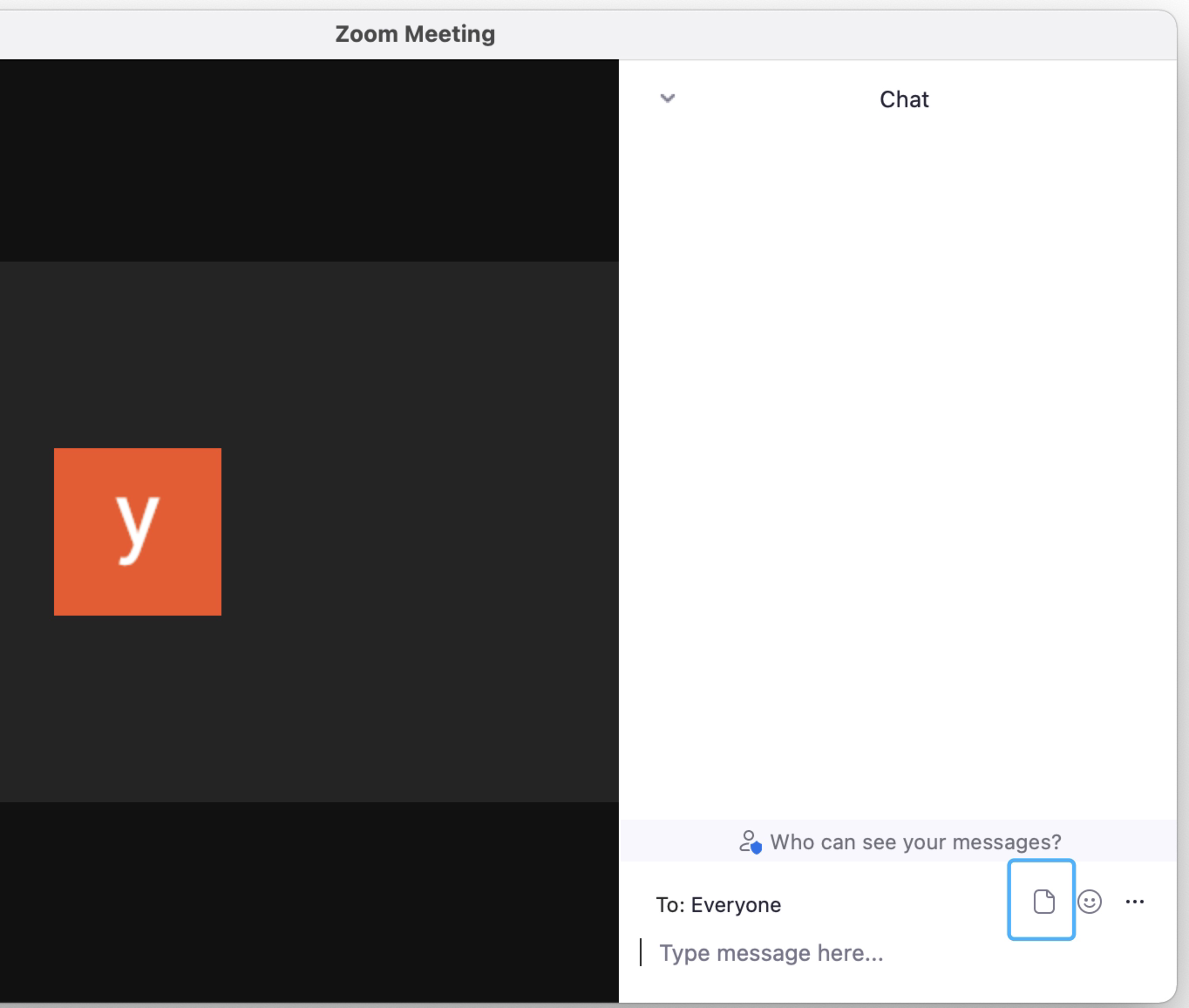


- In Zoom Chat, Click
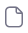
to send the exported zip file to the RA.

| **Create another matrix** |
| --- |

11. You will be creating another matrix during the usability study. You can click on the
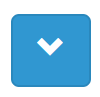
 button to bring back the “Create Matrix” and the “Load Matrix” buttons, and click on ‘Create Matrix’ to create a new matrix.


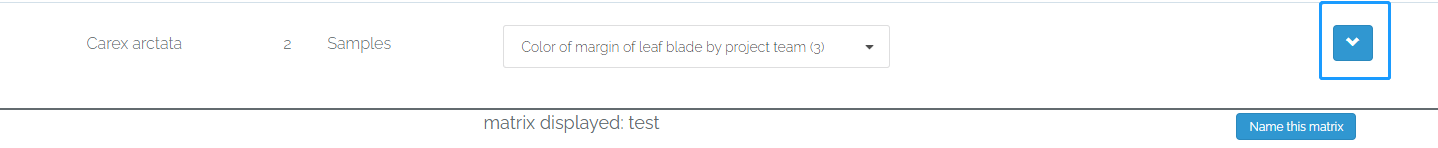


12. You have completed the tutorial. Please feel free to explore a bit more in Character Record until you have a good understanding of how the features work.

| **A quick review** |
| --- |

**The steps for creating and exporting a character matrix:**

1. **Enter taxon name and number of samples**
2. **Select or create all characters needed to describe all samples**
3. **Generate the matrix and enter values in the matrix**
4. **Throughout the process, take the suggestions made by the software and reuse existing characters and values whenever possible.**
5. **Generate one textual description per taxon and export the matrix and description files.**

Character Recorder Usability Student Experiment Task Sheet

**!Welcome to the Usability Study!**

You will complete the tasks on your desktop or laptop computer, not on a tablet or a phone.

**Carefully follow the steps and complete the tasks *correctly* and *quickly*.**

During the process, if you are not sure about certain software features, just do what makes sense to you. The RA has been instructed not to help you with the task. You would inform the RA when you think you have encountered a bug that prevents you from completing the task. To protect your privacy, we are not allowed to record you, your picture, or your voice, therefore please use Chat to communicate with the RA during the session.

| **Get Started**   1. You are now in a Zoom meeting with a RA.    1. Go to ‘Participants’ and change your screen name to your Participant ID.    2. Make sure you are muted and the video is off. Do not share the screen at this time. 2. Complete a demographic questionnaire at <https://uarizona.co1.qualtrics.com/jfe/form/SV_6WpKRtqyrDIUXGe> 3. Now turn on Share Screen in Zoom. **Type** "start the recording" in the chat box to **inform** the RA to start recording. |
| --- |
| **Excel Task**   1. You have selected two samples to record for ***Carex* *muskingumensis*** using your desktop Excel application. Here is the information you will enter for this taxon. Note: phrases connected with ‘or’ below are synonyms. For example, culm and stem are synonyms. You should record either length of culm or length of stem, but not both.   **Sample 1**:  length of culm or stem = 40 cm  Visibility or prominence of vegetative culms = conspicuous  Color or coloration of sheath summits = maroon or reddish brown  shape of sheath summits = U-shaped or U shaped  number or count of spikes = 5  shape of spikes = lancehead-shaped or lanciform  **Sample 2**:  length of culm or stem = 100 cm  color or coloration of sheath summits = brown-red  shape of sheath summits = gutter-shaped  number or count of spikes = 10  shape of spikes = lanceoloid.  5   1. Following the example below, enter the taxon name and the character information for *Carex* *muskingumensis*.   Example:   \| *Carex* *muskingumensis* \| \| \| \| --- \| --- \| --- \| \| Character \| Sample 1 \| Sample 2 \| \| length of clum \| 40 cm \| 100 cm \| \| … \|  \|  \|  1. Save the Excel file and name it *your_PID*.xlsx. 2. **Type** "ready for the next step" in the chat box to **inform** RA when you are ready for the next step. 3. Send the excel file to the RA in Zoom Chat. |
| **Character Recorder Training**   1. Learn to use Character Recorder: <http://shark.sbs.arizona.edu/chrecorder/public/login>    1. Open this [tutorial](https://docs.google.com/document/d/1XAgKAZRYORx06bp_taS5jsOT2KbZ7PaG/edit?usp=sharing&ouid=101696056464925088926&rtpof=true&sd=true) in a new tab. Follow the tutorial to complete a practice exercise.    2. **Type** your password and "ready for the next step" in the chat box to **inform** RA when you are ready for the next step. |
| **Character Recorder Task**   1. Record the same set of characters for *Carex* *muskingumensis* as using Excel, but this time, you will use Character Recorder. Please record the correct information and do not just copy what you did with Excel.    1. Select or create characters and enter values.    2. Name the matrix “experiment”.    3. Create a description for the species.    4. Export the matrix and the description.    5. **Type** "ready for the next step" in the chat box to **inform** RA when you are ready for the next step.    6. Send the exported zip file to the RA in Zoom Chat. |
| **User Experience Questionnaire**   1. **Type** "stop the recording" in the chat box to **inform** the RA to stop the recording and stop sharing the screen. But please keep Zoom open. 2. Complete and submit the questionnaire at <https://uarizona.co1.qualtrics.com/jfe/form/SV_2hHMF68h5ycaRds> 3. **Type** "questionnaire has been completed" in the chat box to **inform** the RA that you have completed the questionnaire. 4. The RA confirms the receipt of the files and the questionnaire result and emails you a $20 Amazon gift card. |

**☺ Thank you for taking part in this study. Your input is valuable to us and to science! ☺**

Appendix D_User Experience Questionnaire_For students and experts

students: <https://uarizona.co1.qualtrics.com/jfe/form/SV_2hHMF68h5ycaRds>

experts:<https://uarizona.co1.qualtrics.com/jfe/form/SV_4PEc6s1NzwWSMWG>


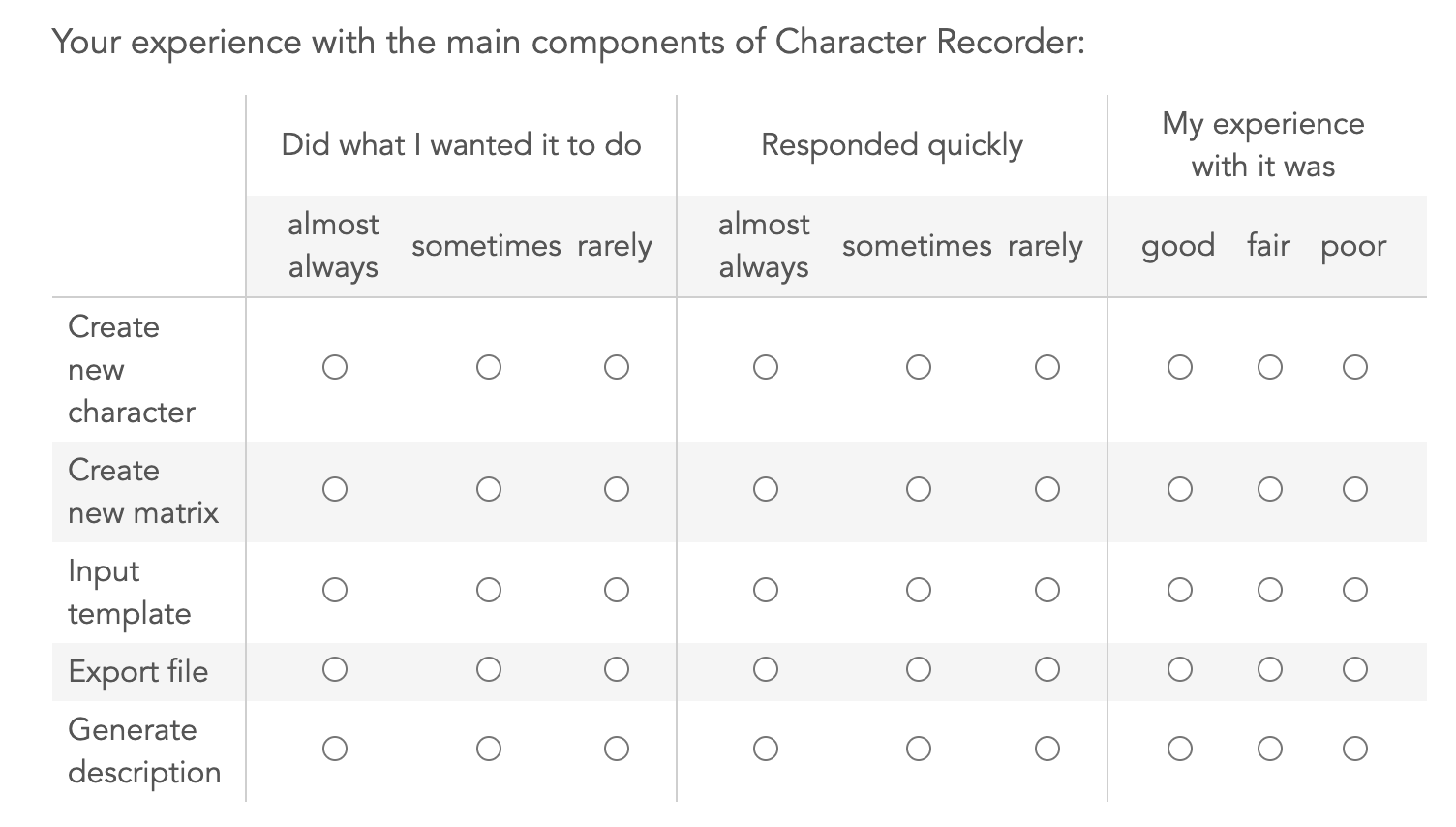


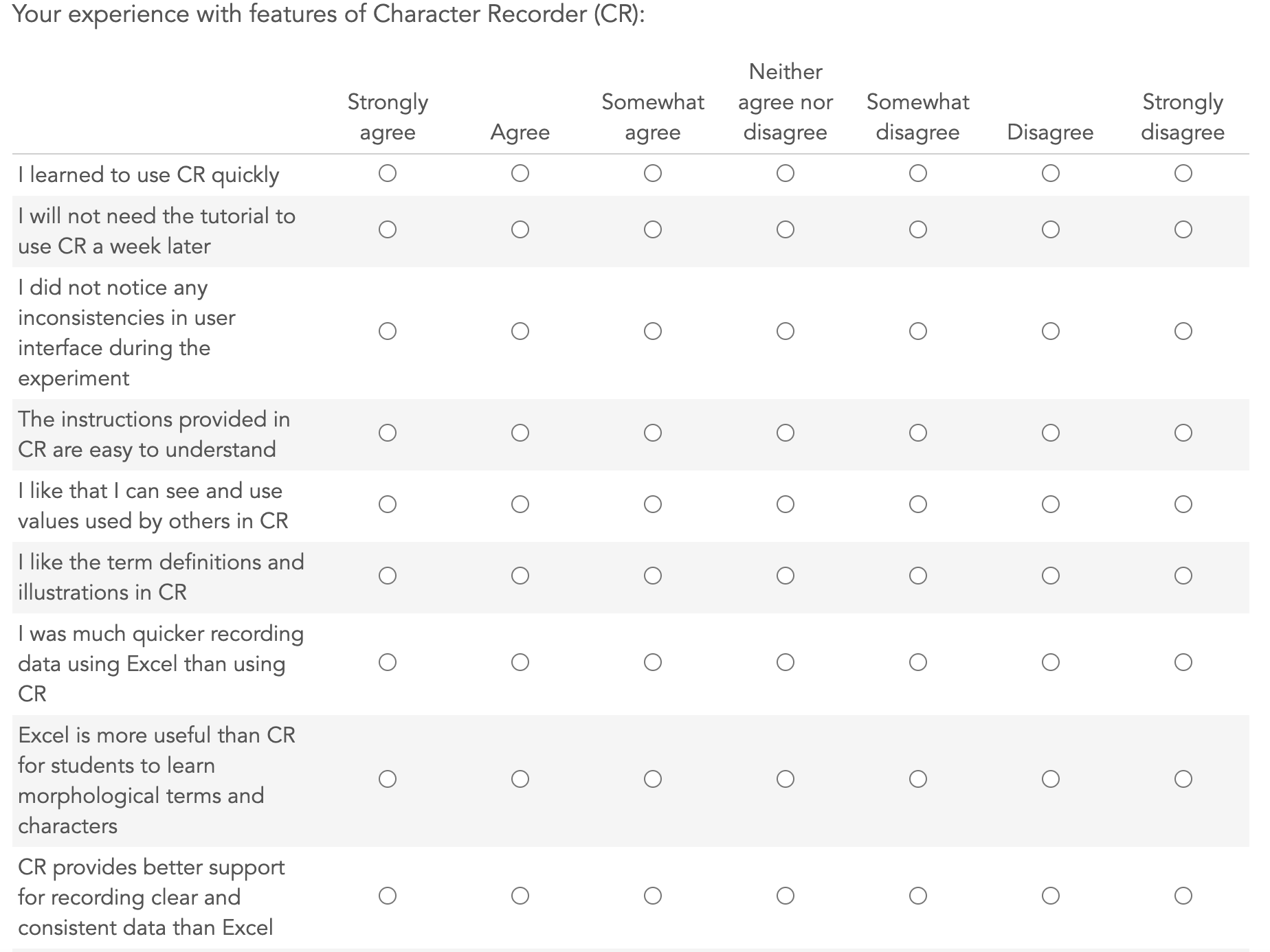


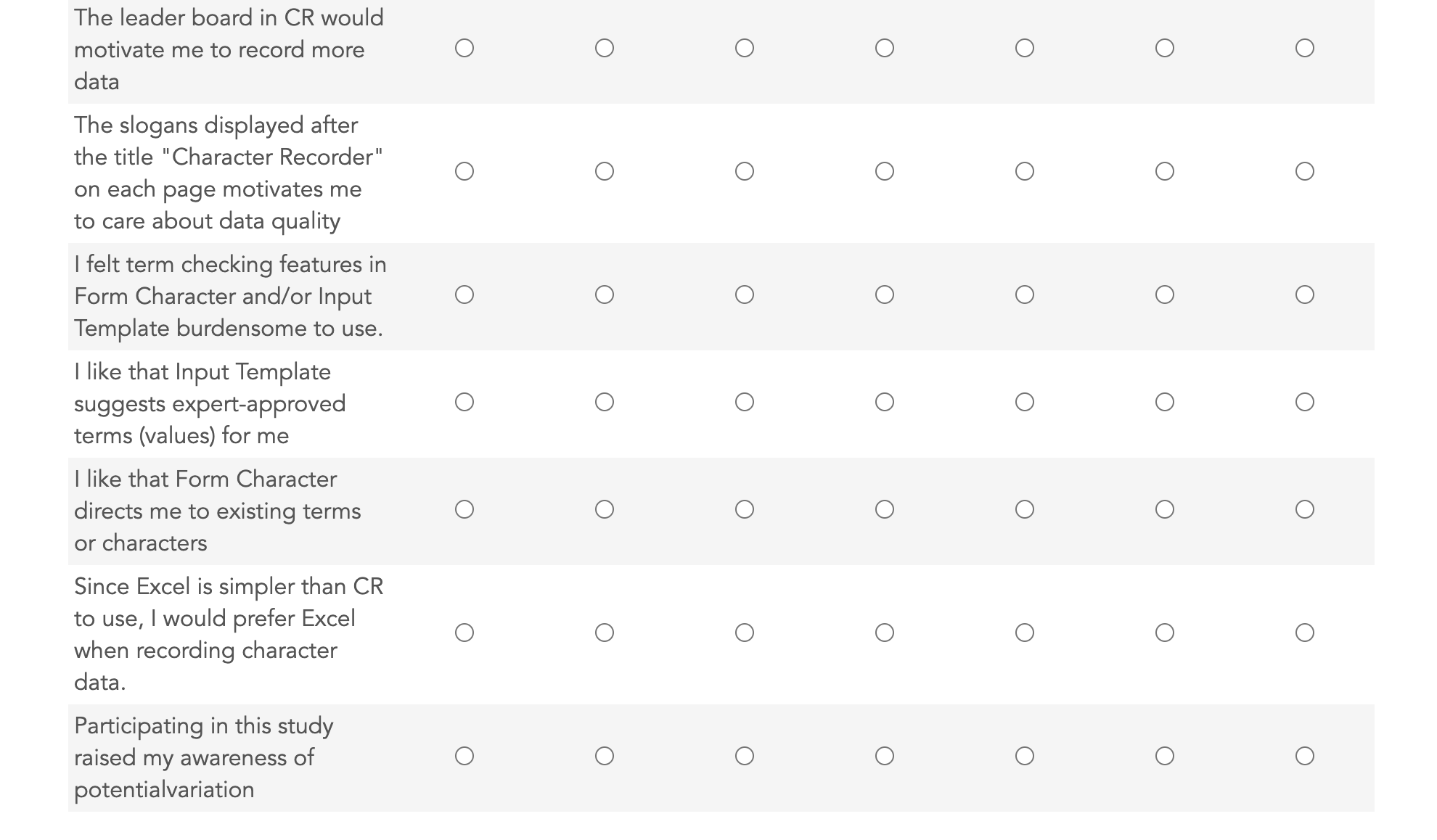


Export’s questionnaire doesn't have “Pariticipating in this study raised my awareness of potentivalvariation”, and add three more question below.


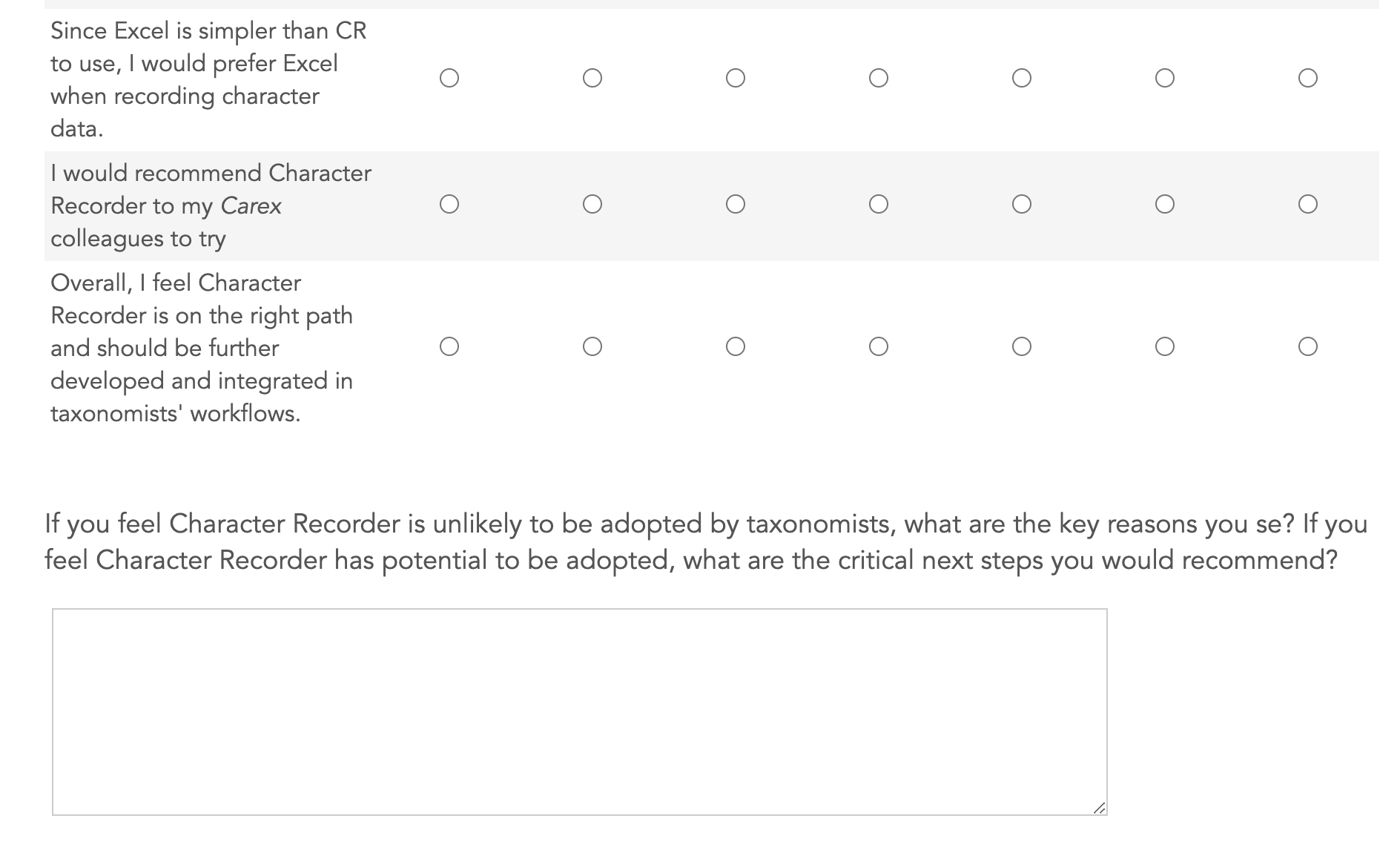


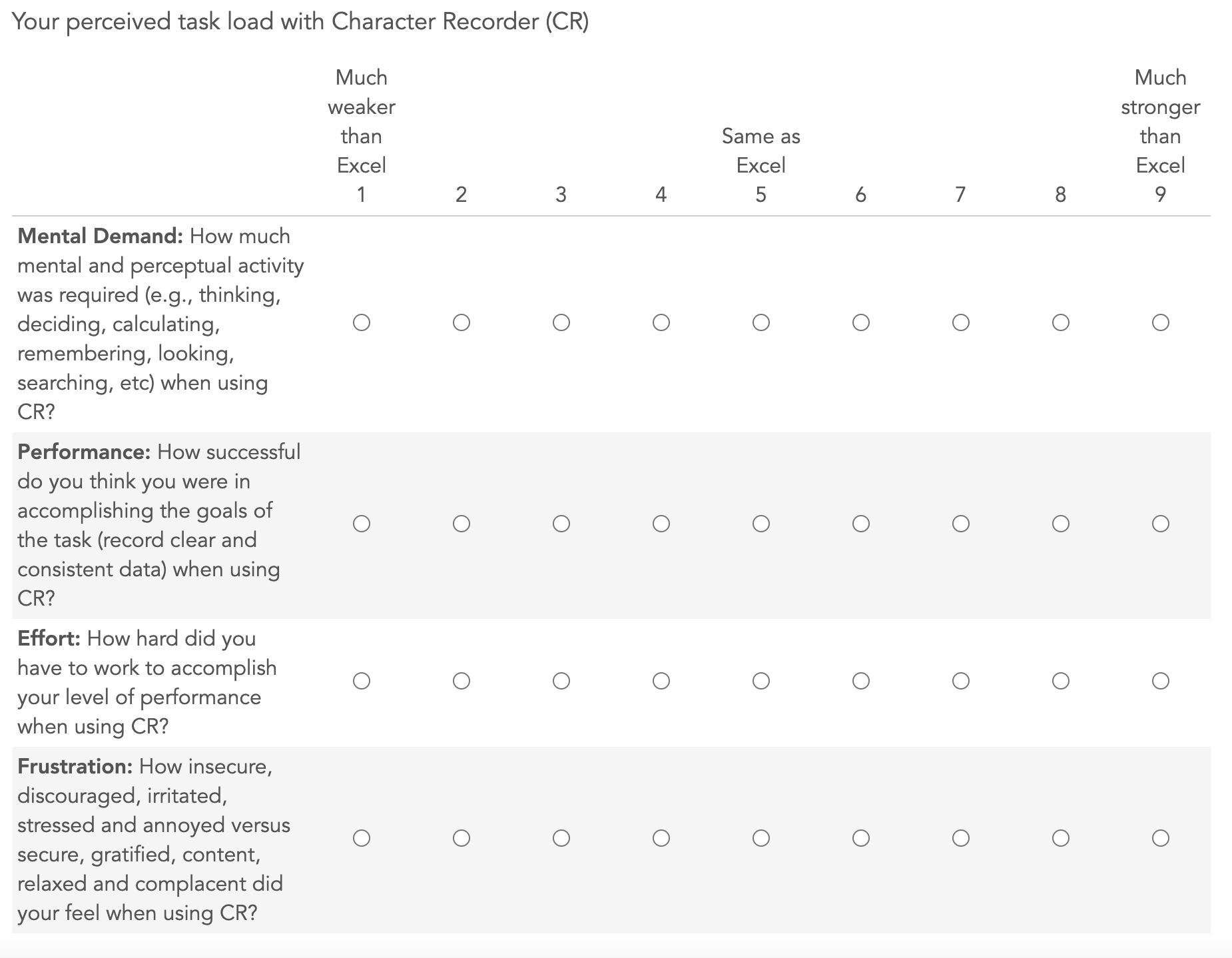


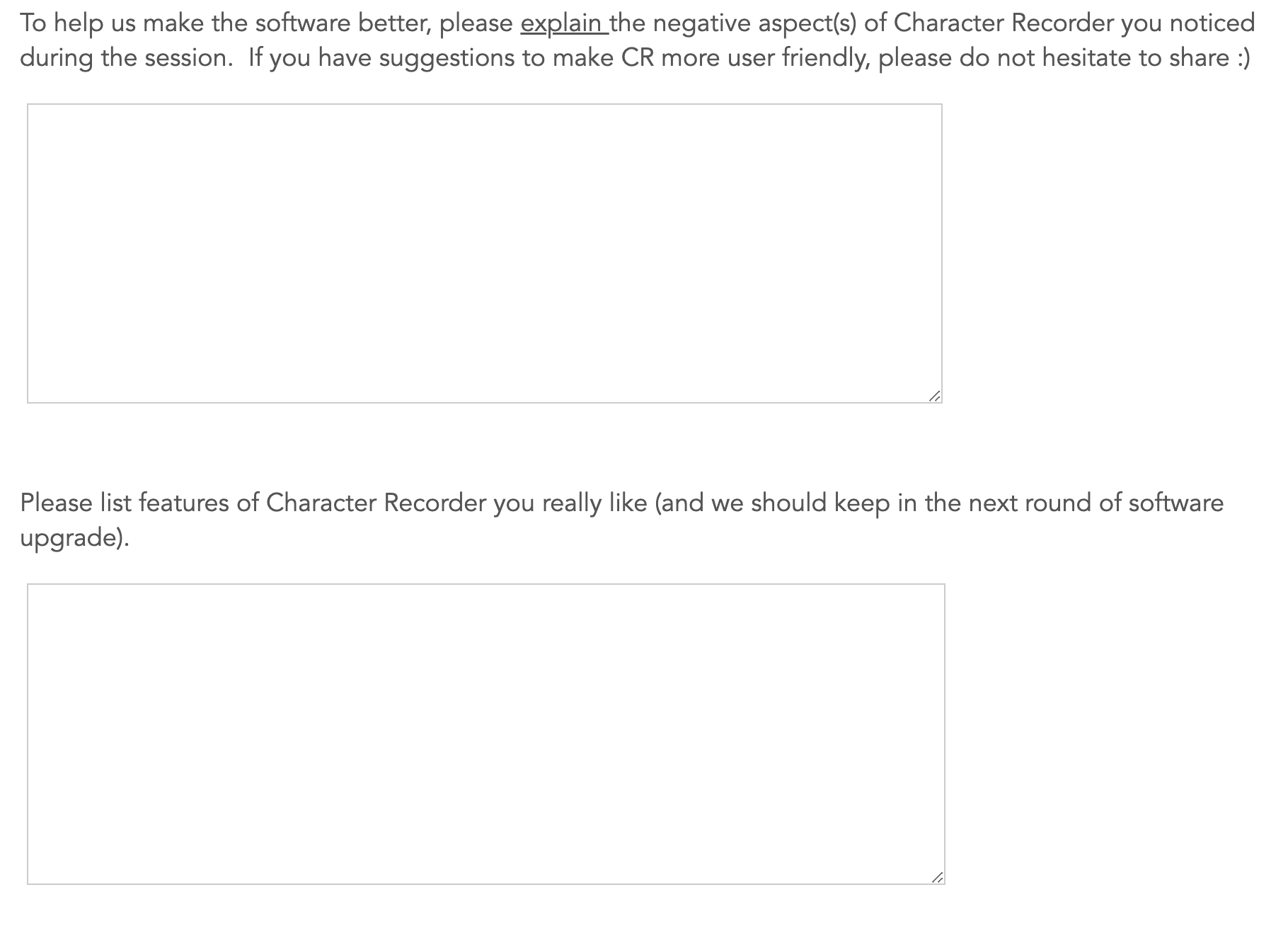


| Q1_1 Your experience with the main components of Character Recorder: Did what I wanted it to do: (N=24) | Almost always | Sometimes | Rarely |
| --- | --- | --- | --- |
| Create new character | 18 | 5 | 1 |
| Create new matrix | 23 | 1 | 0 |
| Input template | 21 | 2 | 1 |
| Export file | 20 | 3 | 1 |
| Generated description | 20 | 4 | 0 |

| Q1_2 Your experience with the main components of Character Recorder: Responded quickly | Almost always | Sometimes | Rarely |
| --- | --- | --- | --- |
| Create new character | 18 | 8 | 0 |
| Create new matrix | 22 | 2 | 0 |
| Input template | 21 | 3 | 0 |
| Export file | 23 | 1 | 0 |
| Generated description | 24 | 0 | 0 |

| Q1_3 Your experience with the main components of Character Recorder: My experience with it was | Good | Faire | Poor |
| --- | --- | --- | --- |
| Create new character | 18 | 5 | 1 |
| Create new matrix | 21 | 3 | 0 |
| Input template | 20 | 4 | 0 |
| Export file | 21 | 2 | 1 |
| Generated description | 19 | 5 | 1 |

| Q2 - Your experience with features of Character Recorder (CR)(N=24) | Strongly agree | Agree | Somewhat agree | Neither agree nor  disagree | Somewhat disagree | Disagree | Strongly disagree |
| --- | --- | --- | --- | --- | --- | --- | --- |
| I learned to use CR quickly | 6 | 15 | 3 | 0 | 0 | 0 | 0 |
| I will not need the tutorial to user CR a week later | 5 | 6 | 9 | 1 | 1 | 1 | 1 |
| I did not notice any inconsistencies in user interface during the experiment | 7 | 11 | 2 | 2 | 0 | 2 | 0 |
| The instructions provided in CR are easy to understande | 8 | 7 | 6 | 0 | 2 | 1 | 0 |
| I like that I can see and use values used by others in CR | 14 | 7 | 2 | 0 | 1 | 0 | 0 |
| I like the term definitions and illurstrations in CR | 9 | 9 | 4 | 0 | 1 | 1 | 0 |
| I was much quicker recording data using Excel than using CR | 3 | 3 | 8 | 6 | 3 | 0 | 1 |
| Excel is more useful than CR for students to learn morphological terms and characters | 0 | 2 | 0 | 1 | 8 | 10 | 3 |
| CR provides better support for recording clear and consistent data than Excel | 8 | 13 | 1 | 0 | 2 | 0 | 0 |
| The leader board in CR would motivate me to record more data | 2 | 4 | 4 | 10 | 3 | 1 | 0 |
| The slogans displayed after the title "Character Recorder" on each page motivate me to care about data quality | 2 | 2 | 7 | 10 | 1 | 1 | 1 |
| I felt term checking features in Form Character and/or Input Template burdensome to use. | 0 | 0 | 7 | 6 | 5 | 5 | 1 |
| I like that Input Template suggests expert-approved terms (values) for me | 7 | 11 | 5 | 1 | 0 | 0 | 0 |
| I like that Form Character directs me to existing terms or characters | 9 | 11 | 4 | 0 | 0 | 0 | 0 |
| Since Excel is simpler than CR to use, I would prefer Excel when recording character data. | 1 | 1 | 2 | 5 | 6 | 5 | 4 |
| (Only student, N=16) Participating in this study raised my awareness of potentialvariation | 6 | 5 | 5 | 0 | 0 | 0 | 0 |
| (Only expert, N=8) I would recommend Character Recorder to my Carex colleagues to try | 2 | 4 | 2 | 0 | 0 | 0 | 0 |
| (Only expert, N=8) Overall, I feel Character Recorder is on the right path and should be further developed and integrated in taxonomists' workflows. | 6 | 2 | 0 | 0 | 0 | 0 | 0 |
| (Only expert, N=8) If you feel Character Recorder is unlikely to be adopted by taxonomists, what are the key reasons you se? If you feel Character Recorder has potential to be adopted, what are the critical next steps you would recommend? |  |  |  |  |  |  |  |

NASA

| Q3 - Your perceived task load with Character Recorder (CR) | Much weaker  than Excel 1 | 2 | 3 | 4 |  | 6 | 7 | 8 | Much  stronger than  Excel 9 |
| --- | --- | --- | --- | --- | --- | --- | --- | --- | --- |
| Mental Demand: How much mental and perceptual activity was required (e.g., thinking, deciding, calculating, remembering, looking, searching, etc) when using CR? | 0 | 0 | 5 | 2 | 4 | 7 | 1 | 5 | 0 |
| Performance: How successful do you think you were in accomplishing the goals of the task (record clear and consistent data) when using CR? | 0 | 1 | 0 | 1 | 2 | 4 | 4 | 6 | 6 |
| Effort: How hard did you have to work to accomplish your level of performance when using CR? | 0 | 2 | 5 | 3 | 3 | 3 | 6 | 1 | 1 |
| Frustration: How insecure, discouraged, irritated, stressed and annoyed versus secure, gratified, content, relaxed and complacent did your feel when using CR? | 0 | 2 | 4 | 5 | 3 | 4 | 4 | 1 | 1 |

Q4:To help us make the software better, please explain the negative aspect(s) of Character Recorder you noticed during the session. If you have suggestions to make CR more user friendly, please do not hesitate to share :)

Q5:Please list features of Character Recorder you really like (and we should keep in the next round of software upgrade).
